# Global Functional Atlas of Escherichia coli Encompassing Previously Uncharacterized Proteins

**DOI:** 10.1371/journal.pbio.1000096

**Published:** 2009-04-28

**Authors:** Pingzhao Hu, Sarath Chandra Janga, Mohan Babu, J. Javier Díaz-Mejía, Gareth Butland, Wenhong Yang, Oxana Pogoutse, Xinghua Guo, Sadhna Phanse, Peter Wong, Shamanta Chandran, Constantine Christopoulos, Anaies Nazarians-Armavil, Negin Karimi Nasseri, Gabriel Musso, Mehrab Ali, Nazila Nazemof, Veronika Eroukova, Ashkan Golshani, Alberto Paccanaro, Jack F Greenblatt, Gabriel Moreno-Hagelsieb, Andrew Emili

**Affiliations:** 1 Banting and Best Department of Medical Research, Terrence Donnelly Center for Cellular and Biomolecular Research, University of Toronto, Toronto, Ontario, Canada; 2 Medical Research Council Laboratory of Molecular Biology, Cambridge, United Kingdom; 3 Department of Biology, Wilfrid Laurier University, Waterloo, Ontario, Canada; 4 Department of Biology and Ottawa Institute of Systems Biology, Carleton University, Ottawa, Canada; 5 Department of Computer Science, Royal Holloway, University of London, Egham, United Kingdom; Johns Hopkins University, United States of America

## Abstract

One-third of the 4,225 protein-coding genes of Escherichia coli K-12 remain functionally unannotated (orphans). Many map to distant clades such as Archaea, suggesting involvement in basic prokaryotic traits, whereas others appear restricted to E. coli, including pathogenic strains. To elucidate the orphans' biological roles, we performed an extensive proteomic survey using affinity-tagged E. coli strains and generated comprehensive genomic context inferences to derive a high-confidence compendium for virtually the entire proteome consisting of 5,993 putative physical interactions and 74,776 putative functional associations, most of which are novel. Clustering of the respective probabilistic networks revealed putative orphan membership in discrete multiprotein complexes and functional modules together with annotated gene products, whereas a machine-learning strategy based on network integration implicated the orphans in specific biological processes. We provide additional experimental evidence supporting orphan participation in protein synthesis, amino acid metabolism, biofilm formation, motility, and assembly of the bacterial cell envelope. This resource provides a “systems-wide” functional blueprint of a model microbe, with insights into the biological and evolutionary significance of previously uncharacterized proteins.

## Author Summary

One goal of modern biology is to chart groups of proteins that act together to perform biological processes via direct and indirect interactions. Such groupings are sometimes called functional modules. The types of protein interactions within modules include physical interactions that generate protein complexes and biochemical associations that make up metabolic pathways. We have combined proteomic and bioinformatic tools, and used them to decipher a large number of protein interactions, complexes, and functional modules with high confidence. In addition, exploring the topology of the resulting interaction networks, we successfully predicted specific biological roles for a number of proteins with previously unknown functions, and identified some potential drug targets. Although our work is focused on E. coli, our phylogenetic projections suggest that a considerable fraction of our observations and predictions can be extrapolated to many other bacterial taxa. As all the data derived from this study are publicly available, others may build on our work for further hypothesis-driven studies of gene function discovery.

## Abstract 3

A novel resource integrating proteomic and genome context-based tools provides a "systems-wide" functional blueprint of*E. coli*, with insights into the biological and evolutionary significance of previously uncharacterized proteins.

## Introduction

Because of its central position in the microbial research community, the Gram-negative bacterium *Escherichia coli* plays a leading role in investigations of the fundamental molecular biology of bacteria [1–8]. This experimentally tractable microbe is a workhorse in basic and applied research aimed at elucidating the mechanistic basis of prokaryotic processes and traits, including those of pathogens. The ever-expanding availability of genomic resources makes E. coli particularly well-suited to systematic investigations of microbial protein components and functional relationships on a global scale. These include a genome-wide collection of single-gene deletion strains [2] along with extensive knowledge of regulatory circuits [3,5,7,9] and metabolic pathways [6,10,11].

Yet despite being the most highly studied model bacterium, a recent comprehensive community annotation effort for the fully sequenced reference K-12 laboratory strains [8] indicated that only half (∼54%) of the protein-coding gene products of E. coli currently have experimental evidence indicative of a biological role. The remaining genes have either only generic, homology-derived functional attributes (e.g., “predicted DNA-binding”) or no discernable physiological significance. Some of these functional “orphans” (not to be confused with ORFans, which are genes present within only single or closely related species) may have eluded characterization in part because they exhibit mild mutant phenotypes, are expressed at low or undetectable levels, or have limited homology to annotated genes. This suggests more-sensitive analytical procedures are warranted.

A key feature of the molecular organization of all organisms, including bacteria, is the tendency of gene products to associate into macromolecular complexes, biochemical pathways, and functional modules that in turn mediate all the major cellular processes. Elaboration of these interaction networks via proteomic, genomic, and bioinformatic approaches can reveal previously overlooked components and unanticipated functional associations [12]. For example, a recent integrative analysis of phenotypic, phylogenetic, and physical interaction data led to the discovery of an evolutionarily conserved set of novel bacterial motility-related proteins [13]. However, although systematic integration of diverse high-throughput interaction datasets is routinely performed to reveal new functional relationships in model eukaryotes such as yeast, worm, and fly [14–19], few analogous studies of the global functional architecture of E. coli, and any prokaryote for that matter, have been reported to date [20–22].

To this end, we have combined complementary, highly sensitive computational and experimental procedures to derive extensive high-quality maps of the functional interactions inferred by genomic context (GC) methods and physical interactions (PI) deduced by proteomics of E. coli. Our results indicate that many previously unannotated bacterial proteins are components of functionally cohesive modules and multiprotein complexes linked to well-known biological processes. A substantive fraction of these associations could be verified by independent experimentation and were found to be broadly conserved across prokaryotic phyla, indicating homologous systems in other microbes, whereas others are seemingly restricted to the E. coli lineage. The entire data collection is publicly accessible via a searchable Web-browser interface to stimulate exploration of both conserved and specialized bacterial proteins within the context of biological processes of particular interest.

## Results

### The Extent of Existing Functional Annotation for E. coli Proteins

**Figure 1:**
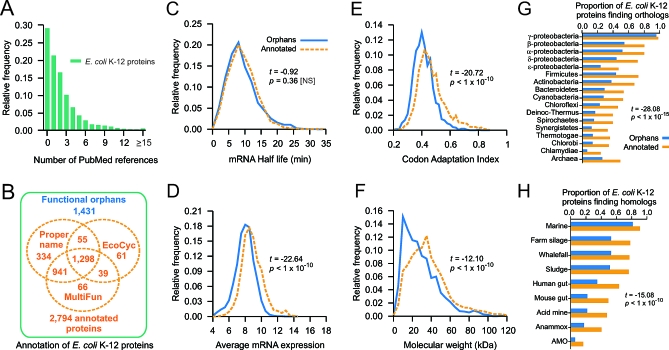
Annotated and Functional Orphan Genes of the E. coli K-12 Reference Strain (A) Frequency distribution of supporting publications per E. coli protein-coding gene. (B) Summary of existing annotations for E. coli, showing proteins of unknown function (orphans) lacking proper names and functional annotations in MultiFun [25] or EcoCyc [11]. (C–F) Although the functional orphans are encoded by transcripts with half-lives comparable to those of annotated genes, they tend to be expressed at lower levels based on (D) microarray analysis of mRNA and (E) CAI scores, and (F) have lower molecular weights on average. The *x*-axis in (D) represents the average of the log-scale mRNA expression level of each gene for all the arrays using the Robust Multi-Array Average normalized data obtained from the M3D database [103]. NS, not statistically significant; *p*, *p*-value; *t*, *t*-test. (G) Orthologs of orphans are also less prevalent in sequenced genomes than those of annotated genes. (H) However, examination of environmental metagenomic libraries indicates that the orphans are not necessarily exclusive to the *Escherichia* lineage. *Anammox*, anaerobic ammonium oxidation bacteria; *AMO*, methane oxidizing Archaea.

Since the functional characterization of E. coli, and bacteria in general, has largely been guided historically by scientific interests and technical considerations, some bias is expected in terms of the coverage and depth of existing biological knowledge as reflected in current gene annotations. To evaluate the degree to which the physiological functions of the 4,225 putative protein-coding sequences of E. coli K-12 are characterized presently, we examined the scope of literature reference records curated in the UniProt annotation system [23]. After excluding PubMed references corresponding to genomic mapping studies, the average total number of papers associated with each of the proteins of E. coli K-12 is surprisingly limited (Figure 1A), with many proteins apparently still uncited.

We next examined recent E. coli K-12 (substrains W3110 and MG1655) gene annotations in the public databases RefSeq [24], MultiFun [25], and EcoCyc [11]. Since W3110 is commonly used for high-throughput studies, we devoted the bulk of our subsequent analysis to this substrain. However, to make sure that relevant functional attributes were not overlooked, we cross-mapped the corresponding gene accessions in both substrains and compiled an inclusive set of functional annotations accordingly (Table S1). In total, we found that 2,794 (66%) of E. coli's proteins had either proper mnemonic names [26], experimentally derived annotations in the MultiFun multifunction schema, or literature documentation to a well-defined pathway or multiprotein complex in EcoCyc (Figure 1B). This left 1,431 proteins (34%) as currently functionally uncharacterized (which constitute our orphans set, listed in Table S1; see Protocol S1 for details). Of these, 446 (31%) have at least one putative molecular function defined on the basis of sequence (such as the presence of a predicted DNA-binding domain or an enzymatic motif) in the Clusters of Orthologous Groups (COGs) of proteins catalog [27].

### Properties of the Functional Orphans of E. coli

The genes lacking annotation appear to be translated into bona fide proteins as their corresponding transcripts [28] were not significantly (*p* = 0.36) less stable than the products of annotated genes (Figure 1C). However, some differences were evident in terms of their biophysical attributes and evolutionary scope relative to annotated genes (Table S1). Most notably, only 21 orphans (1.5%) are required for viability under standard laboratory conditions [2] in contrast with the 280 annotated genes (10%) previously deemed essential. The orphans were also significantly (*p* < 1e−10) less abundant at both the transcript (Figure 1D; average normalized mRNA expression over 400 microarray experiments [5]: 8.0 [orphans] vs. 8.9 [annotated]) and protein levels (Figure 1E; average codon adaptation index [CAI]: 0.41 vs. 0.47). Furthermore, they tend to encode somewhat smaller proteins (Figure 1F; average *M_W_*: 29.4 vs. 38.2 kDa; *p* < 1e−10) with fewer domain assignments (44%) than for annotated proteins (74%) according to the SUPERFAMILY database [29] (Table S1).

Orphans also generally find fewer orthologs in a nonredundant genome dataset, filtered at 90% similarity based on the frequency of shared orthologs (Figure 1G), with an average of 0.22 as compared with 0.48 for annotated genes (*p* < 1e−10) using a maximum-score E-value cutoff of 1 × 10^−6^ for BLAST bidirectional best hits (BDBHs; Table S2 and Protocol S5 for details). Nevertheless, broader sequence comparisons against currently available metagenomes (Figure 1H) indicated that orphan homologs (one-way BLAST hits) are often widely distributed in diverse environments (Table S2 and Protocol S2); for example, a high proportion (0.80) of orphans have homologs present in marine metagenomes, anaerobic bacterial populations (farm silage, 0.51; whalefall, 0.50; sludges, 0.49), and even in the residents of the mammalian gut (union of human and mouse, 0.35), implying participation in core bacterial processes. Furthermore, the same high proportion (∼99%) of orphan and annotated genes have orthologs in the other sequenced E. coli isolates, including pathogenic variants and closely related *Shigella* strains (Table S2). Taken together, this argues that the functional significance of the orphans is more pervasive than the current annotations suggest.

### A Systematic Approach to Elucidate Biological Function

**Figure 2:**
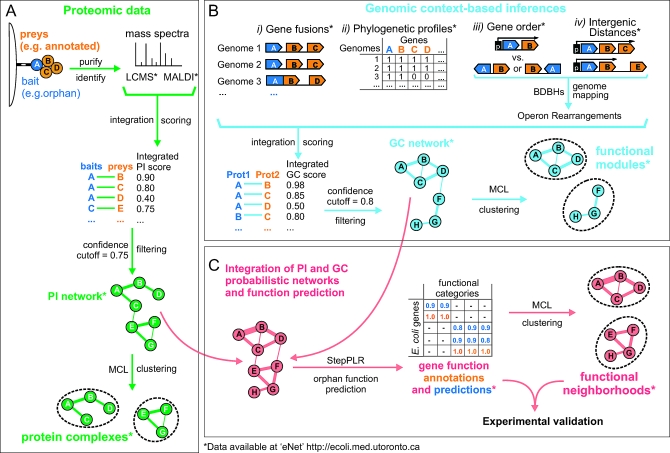
Generation and Integration of Physical and Functional Networks and Orphan Function Prediction (A) Construction of a PI network based on protein copurification and detection by mass spectrometry. The confidence scoring of the LCMS and MALDI networks was conducted using a logistic regression with datasets consisting of PI from low-throughput studies curated in DIP, BIND, and IntAct (gold positives) and proteins in different subcellular localizations (gold negatives). The two networks were integrated using a probabilistic model [61] (Protocol S6). The resulting PI network, with edge weights corresponding to likelihood ratios, was clustered using MCL to delimit “multiprotein complexes.” (B) Integration of four GC methods into a single functional interaction network using the same probabilistic model [61] and resulting scores (edge weights) were input to MCL to delimit “functional modules.” (C) Orphan function prediction was conducted using a “guilt-by-association” procedure. After integration of PI and GC interactions into a single probabilistic network [61], a machine learning algorithm (StepPLR) newly developed for this study was used to assign functions based on the binary associations of orphans with annotated proteins, the respective interaction edge weights, and the overall network topology. Correlations between vectors of these function predictions (orphans), and the annotations were then used as input to delimit “functional neighborhoods” by clustering using MCL.

The scarcity of the existing knowledge regarding the biological roles of the orphans is likely due to multiple reasons, ranging from the lower expression, nonessentiality, or smaller sizes of certain orphan proteins to their lack of obvious homologs in other organisms including humans. Accordingly, integration of multiple data sources is warranted to decipher the specific biological roles of this uncharacterized repertory. Since the elucidation of physical and functional interaction networks can provide insights into bacterial protein function based on the concept of guilt by association [30], we took a multipronged approach. We performed large-scale proteomic analysis to determine orphan participation as components of multimeric protein complexes, and inferred functional relationships based on genomic context inference, which exploits the patterns of gene conservation across bacterial genomes [31]. We then predicted the functions of the orphans using an integrative machine-learning procedure with extensive benchmarking. Finally, we performed independent experiments to validate a subset of high-confidence predictions related to core biological processes. Key steps in our pipeline are outlined schematically in Figure 2.

### Experimental Definition of the Physical Interaction Network of the Soluble Proteome

We performed systematic large-scale tandem-affinity purifications of all endogenous soluble orphan and annotated proteins detectably expressed in E. coli W3110 under standard culture conditions (see Materials and Methods and Protocol S3 for details). We used an optimized Sequential Peptide Affinity (SPA)-tagging system to isolate multiprotein complexes [32]. This procedure is based on the integration of a marker cassette bearing a dual-affinity tag, consisting of three FLAG sequences and a calmodulin-binding peptide separated by a protease cleavage site, fused to the C-termini of targeted open reading frames in E. coli DY330 (W3110 background) via λ-phage “Red”–mediated homologous recombination. This system enables recovery of native bacterial protein complexes at near-endogenous levels [4], minimizing spurious nonspecific protein associations. Stably interacting polypeptides were subsequently detected using a highly sensitive combination of tandem mass spectrometry (LCMS) and peptide mass fingerprinting procedures (MALDI) to increase detection coverage and accuracy (Protocol S3), just as we had previously done in a focused investigation of highly conserved essential E. coli proteins [4]. We successfully chromosomally tagged 1,241 new baits, aiming to verify putative interactions by reciprocal tagging where possible, for a total of 1,476 large-scale protein purifications (after including the 235 reported previously), of which 552 represented orphans (Protocol S3).

Since proteomic datasets typically contain noise in the form of nonspecific associations, we performed a careful statistical analysis and quality filtering to determine biologically meaningful PI. We considered that the specificity and affinity between any two putatively interacting proteins should be correlated with the consistency of copurification over all the experiments in which the proteins were identified (i.e., co-complexed). We therefore used an established copurification metric [33] to assess interaction specificity based on the similarity of the protein copurification patterns (Protocol S3). We then generated a single consolidated confidence score for each putative pairwise physical interaction based on the copurification metric together with the primary interaction evidence to penalize inconsistent or promiscuous binders (i.e., possible false positives) using alternatively a logistic regression model and Bayesian inference [34] (Protocol S3).

The logistic regression model was trained using a reference set of curated gold-standard PI (Protocol S3), which represents the union of experimentally verified PIs derived from low-throughput experiments extracted from the Database of Interacting Proteins (DIP) [35], the Biomolecular Interaction Network Database (BIND) [36], and the IntAct database [37]. For the negative gold standards, we compiled pairs of proteins annotated with different subcellular localizations (i.e., one cytoplasmic, the other periplasmic or outer membrane-bound [38].

**Figure 3:**
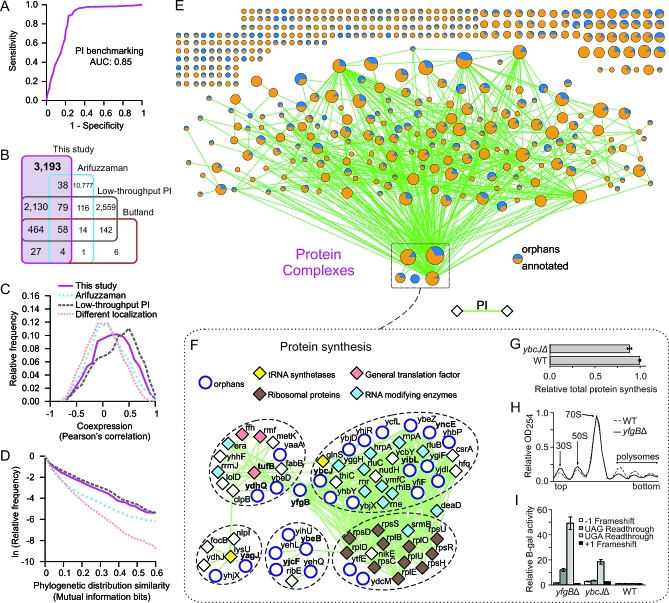
High-Confidence Physical Interactions and Putative Multiprotein Complexes (A) Benchmarking of the experimentally derived PI network in E. coli against positive and negative gold standards by receiver operating characteristic (ROC)-curve analysis; cumulative area-under-the-curve (AUC) is shown as an overall performance measure. (B) Overlap of PI identified in this study with previous proteomic reports [1,4] and low-throughput PI obtained from DIP, BIND, and IntAct. (C and D) Putatively interacting proteins have highly correlated gene expression patterns (C) and similar phylogenetic profiles (D) based on mutual information as for low-throughput curated PI and in contrast to control protein pairs derived from different subcellular compartments. (E) Graphical schematic of putative stable, soluble multiprotein complexes, drawn using the GenePRO Cytoscape plugin [104] (see Table S7 for listing). Each node represents a complex, whose size reflects the number of contained proteins; edge widths reflect the number of interactions between subunits of different complexes. (F) Multiprotein complexes implicated in the bacterial translation apparatus; orphan and annotated genes mentioned in the main text are highlighted in bold. (G) Reduced rate of total protein synthesis in a strain lacking *ybcJ* relative to wild-type cells (WT). (H) Perturbed ribosome profiles in an *yfgB* deletion strain. (I) Elevated rates of frame-shifting and stop-codon readthrough in *yfgB* and *ybcJ* deletion strains relative to wild-type (WT). β-gal activity is only produced after the corresponding translational defect has occurred; error bars indicate standard deviation.

Despite its relative simplicity, the logistic regression model offered better performance than the Bayesian method (see Figure 3A and Table S3). We therefore applied the former to our global PI network, assigning a probabilistic confidence score for each pair of putatively interacting proteins (Protocol S3). To minimize false positives without incurring excessive false negatives, we further filtered our network using a stringent minimum confidence cutoff of ≥0.75 as a high proportion (71%) of PI verified by reciprocal purification (Table S4) had likelihood scores at or above this threshold (Figure S1A). Finally, we removed the ten most-highly connected “hub” proteins that were deemed particularly abundant nonspecific contaminants (Table S5).

The resulting final network consisted of 5,993 high-confidence, nonredundant pairwise interactions among 1,757 distinct E. coli proteins, including 451 orphans, or roughly two-thirds of the predicted soluble cytoplasmic proteome. As summarized in Figure 3B, most (3,193, or 53%) of these PI are novel (Table S6), whereas only 47% were already reported in either the DIP, BIND, or IntAct interaction databases, or previous large-scale proteomic studies [1,4]. Importantly, our filtered dataset had a comparable level of accuracy (median confidence of 0.79) as for the much smaller set of 716 “validated” PI previously reported by our group [4] and a genome-scale dataset of 7,123 PI (median confidence of 0.69) generated using an analogous affinity purification schema in yeast [39].

The reliability of our dataset was also evident by two additional independent criteria. First, the mRNA expression patterns of the putatively interacting proteins were nearly as highly correlated as those of PI determined by low-throughput experiments (Figure 3C), even when these last are formed by presumably more abundant proteins (Figures S1E and S1F). Second, despite the more limited evolutionary distribution of the orphans, the putatively interacting proteins exhibited an elevated degree of co-occurrence of the respective orthologs across other bacterial species, evident in the high mutual information of the corresponding phylogenetic profiles (Protocol S5), again comparable to that of interacting pairs derived from low-throughput experiments (Figure 3D).

Collectively, these results indicate that our physical interaction network is very likely to be informative about orphan protein function.

### Orphan Membership within Multiprotein Complexes

Since macromolecular assemblies mediate biological function in cells, we partitioned our high-confidence physical interaction network using the Markov clustering algorithm (MCL; see Materials and Methods and Protocol S4) to define orphan membership as subunits of discrete multiprotein complexes. MCL simulates random walks (i.e., flux) to delimit highly connected subnetworks based on both the connectivity and the weight of the graph edges [40]. In this case, the weights reflect the integrated PI scores obtained by logistic regression (Figure 2A). The higher the flux within in a region of the PI network, the more likely MCL will delimit the region as a cluster (in this case, a putative multimeric protein complex). A recent comparative study [41] found that MCL is often superior to other clustering algorithms in identifying functionally related groupings in probabilistic molecular interaction graphs and is remarkably resilient to spurious graph perturbations (e.g., missing edges).

We optimized the MCL parameters (see Materials and Methods and Protocol S4) to partition the 5,993 PI network, generating a set of 443 putative multiprotein complexes (Figure 3E), most of which consist of two to four polypeptides (Table S7). In agreement with previous reports [41], alternative clustering algorithms comparable to MCL in terms of accuracy, such as the Restricted Neighborhood Search Cluster algorithm [42], produced similar groupings (unpublished data). Moreover, as was found in a proteomic survey of yeast multiprotein complexes [39], both the subunit number and degree of connectivity of the MCL clusters followed a power-law distribution (Figure S2B and S2C). More telling, 244 (55%) of these E. coli multiprotein complexes contained at least one orphan as a putative subunit, with linkages suggestive of concerted biological functions (Figure 3E). The complexes also showed a significant (*p* < 0.001) enrichment in terms of functional homogeneity, compared with null random models (Figure S2A), implying that both the annotated components and the associated orphans tend to participate in the same biological processes.

For example, 25 orphans were detected as part of a large subnetwork of putative complexes involved in protein synthesis (Figure 3F). These include the orphans YbcJ and YncE, which physically interacted with the pseudouridylate synthase RluB, the RNA helicases SrmB and DeaD, the exoribonucleases E (Rne) and R (Rnr), and other components of the ribonucleolytic “degradosome” responsible for mRNA degradation, suggesting a probable role in RNA processing and/or turnover. Likewise, YfgB copurified with three translation-related complexes, including ribosomal proteins. Consistent with these observations, the expression of YncE, which has similarity to the nonribosomal peptide synthase AfuA of Aspergillus fumigatus, is reduced more than 9-fold upon exposure of E. coli to the translational inhibitor puromycin [43]. We also determined that deletion of *ybcJ* results in a significant reduction in the incorporation of ^35^S-labeled methionine in vivo relative to wild type (Figure 3G), indicating a decrease in the global rate of protein synthesis. Similarly, ribosome profile analysis (Figure 3H) showed that inactivation of *yfgB* decreased the level of mature polysomes actively engaged in mRNA translation and altered the cellular ratios of 30S and 50S ribosomal subunits relative to 70S monosomes. Moreover, both the *ybcJ* and *yfgB* mutants exhibited reduced translation fidelity (Figure 3I) as assayed by four reporter plasmids that measure the frequency of frameshifts and stop codon readthrough.

Other orphans in this translation subnetwork include YibL, which copurified both with YfgB and YbcJ, and with RNA processing factors involved in ribosome biogenesis, such as the RNA pseudouridine synthetases RluB/RluC and the RNA helicase DeaD, and with RppH (formerly NudH), which was recently identified as a regulator of 5′-end–dependent mRNA degradation [44–46]. Similarly, the orphan YdhQ copurified with translation elongation factor Tu, whereas YagJ interacted with lysine tRNA synthetase (LysU); and YjcF, which has similarity to phenylalanyl-tRNA synthetase PheT of Bacteroides vulgatus, bound ribosomal release factor 2 and another orphan, YbeB, which in turn was found to associate with the 50S ribosome subunit, as recently reported [47]. These results confirm that our high-confidence physical interaction network is informative about the function of at least certain orphans.

### Functional Interactions Predicted by Genomic Context Methods

Although we attempted to tag and purify the entire soluble E. coli interactome, we failed to detect 469 orphan proteins by MALDI or LCMS, presumably because they are membrane-associated (∼35%; Figure S1B) and hence not soluble, or are of particularly low abundance (∼40%), as reflected by their CAI and mRNA levels (Figures S1C and S1D, respectively). To bypass this limitation, we applied computational methods to discern a network of high-confidence pairwise functional interactions for all E. coli proteins, including those not detectable by proteomic methods, by examining the natural chromosomal clustering of bacterial genes. As illustrated in Figure 2B, we used four different GC methods, namely: (1) gene fusions [48,49]; (2) similarity between phylogenetic profiles [27,50,51]; (3) evolutionary conservation of gene order [52–54]; and (4) intergenic distances [55–57] (see Materials and Methods and Protocol S5 for details). The latter two methods are independent approaches to detect operons and their subsequent rearrangements across prokaryotic genomes. In particular, the intergenic distances method, leads to considerably more high-quality predicted functional associations compared with the first three classic GC methods [55], and does not depend critically on the detection of orthologs in evolutionarily distant genomes, making it potentially better suited for detecting functional interactions involving orphans.

**Figure 4:**
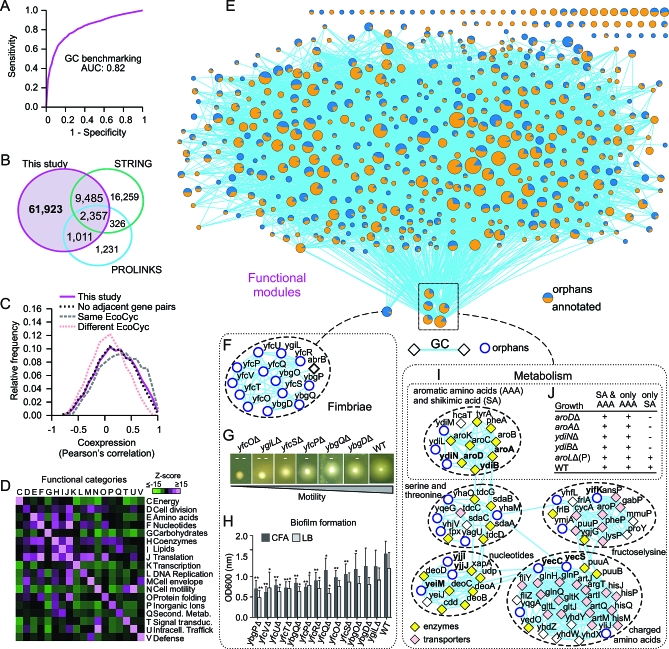
High-Confidence Genomic Context Associations and Putative Functional Modules (A) Benchmarking of unified GC interactions in E. coli against positive and negative gold standards by ROC-curve analysis. (B) Overlap of high-confidence functional interactions predicted in this study with two other public GC databases. (C) Even after eliminating adjacent gene pairs to control for known and predicted E. coli operons, functionally linked genes have highly correlated patterns of mRNA expression comparable to components of the same curated EcoCyc pathways rather than different pathways. (D) Functionally linked genes are enriched for annotations to the same COG functional categories. (E) Graphical representation of putative E. coli functional modules (see Table S9 for listing); node size and colors are proportional to the number and fraction of orphan and annotated subunits, respectively, while lines represent interactions connecting modules. (F) Putative fimbriae-related module. (G) Defective motility of mutant strains deleted for orphans linked to fimbriae (from [F]); single dashes (-) indicate moderately impaired motility, while double dashes (–) represent strong repression. Other mutants displaying a normal phenotype comparable to the wild-type strain BW25113 (WT) are not shown. (H) Defective biofilm formation by mutants deleted for fimbriae-related orphans (from [F]); significant differences (*t*-test) in cell adhesion (absorbance) between mutant and WT strains are denoted by asterisks (single asterisks [*], *p* < 0.01; double asterisks [**], *p* < 0.0001). Error bars indicate standard deviation of the mean. CFA, colonization factor antigen medium; LB, Luria Bertani medium. (I) Metabolic modules mentioned in main text. (J) Mutants auxotrophic for aromatic amino acids show defective growth on minimal media containing shikimic acid. A prototroph—*aroL*Δ mutant strain (P)—is shown for comparison.

The pairwise interactions generated by each of these prediction methods were independently evaluated by benchmarking using suitable gold standards. Positive gold standards were defined as pairs of E. coli genes belonging to the same biological pathway as defined in EcoCyc, while the negative gold standards represented pairs of annotated E. coli genes whose products participate in different pathways (see Protocol S5 for details). The results of each GC method were subsequently combined to create a single unified functional association score (Protocol S6 and Figure 2B). Although different data integration algorithms have been developed [58–61], most of these have a similar probabilistic basis and assumptions. For this study, we opted for the integration procedure used by von Mering and colleagues [61] to construct the Search Tool for the Retrieval of Interacting Genes/Proteins (STRING) database. This approach treated the reliability of the associations generated by each GC method as independent probabilities, such that the likelihood of an interaction is proportional to the number of times it was observed and the degree to which each GC method contributed to the overall network reliability (Protocol S6). Finally, we applied a stringent filter to the unified functional network to obtain a set of 74,776 high-confidence (probabilities ≥0.80) nonredundant interactions (Figure 4A and Table S8).

Despite the tendency of the orphans to exhibit more limited conservation notwithstanding the dependency of GC methods on homologs in multiple species (except for operon predictions based on intergenic distances [55]), our combined GC network implicated virtually all (1,367, or 96%) of the orphans in 23,365 pairwise functional interactions (Table S8). Moreover, relatively few (<18%) of our predicted interactions appear to have been reported previously (Figure 4B). Although we could not meaningfully compare our results to an alternate set of putative functional links generated recently [22] because of a lack of publicly accessible dataset scores, we found that less than 5% (3,368) of our predicted interactions are listed in the PROLINKS comparative genomics databank [62], whereas only approximately 16% (11,842, of which only 2,613 involve an orphan) were found in STRING (v. 7.1) at a more liberal 0.7 confidence threshold. More critically, greater than 85% of our predicted orphan interactions involve a functionally annotated E. coli protein, indicating a good potential to make functional inferences. The fact that PROLINKS has 1,657 predictions not attained by our integrative approach may reflect our use of a higher confidence threshold as well as differences in implementation of the GC measures and the identification of putative orthologs. For instance, whereas we used BLAST-BDBHs as criteria to detect orthologs between pairs of genomes, STRING uses COG-based definitions of orthology, whereas PROLINKS uses one-way BLAST hits (not necessarily orthologs). Conversely, most of the 16,585 predictions exclusive to the STRING database were compiled using text mining or alternate experimental criteria such as protein–protein interactions, whereas the highest numbers of predictions exclusive to our GC datasets come from operon rearrangements.

The reliability of our unified functional association network was independently corroborated based on the high correlations of expression among putatively interacting gene pairs (Figure 4C), which was comparable to that observed for components of the same curated EcoCyc pathway even after eliminating all pairs of genes belonging to an experimentally characterized operon or all adjacent gene pairs in E. coli (Figure 4C). We also observed a marked enrichment for interactions among proteins annotated to the same curated functional categories (Figure 4D), implicating by extension any associated orphans in these same processes.

### Defining the Participation of Orphans as the Components of Functional Modules

Groups of functionally interacting genes form functional modules centered on a common process or biochemical pathway(s). To define orphan participation as components of such modules, we partitioned the high-confidence GC network using MCL (Protocol S4), generating a total of 507 putative functional modules consisting of two or more components (Figure 4E and Table S9). Examination of the functional homogeneity of these predicted modules (see Materials and Methods and Protocol S4) indicated, as for our putative multiprotein complexes, that they were highly enriched (*p* <0.0001) for concerted annotated biological processes (Figure S2D), again implicating the associated orphans in these same roles. Module membership followed a characteristic power law distribution (Figure S2E) with most modules having between two and 10 components, but the overall node connectivity did not (Figure S2F); further analysis is necessary to determine the significance of this divergent behavior.

Two hundred and eighty-nine (57%) of the modules had at least one of a total of 1,189 different orphans. One notable example is shown in Figure 4F. Diverse lines of experimental and bioinformatic evidence support the involvement of this putative module in the biogenesis and/or activity of fimbriae, appendages or pili that are shorter than the characteristic flagellum of Gram-negative bacteria, which mediate cell adhesion, biofilm formation, motility, and host invasion [63,64]. For instance, 12 of the 13 orphan components possess sequence characteristics of bacterial adhesins and chaperone/Usher pili protein families [29,65]. Gene expression profiling studies [66,67] have previously established that most of these orphans are also coordinately induced during biofilm formation (Table S10). Perhaps most compellingly, we found that single-gene E. coli knockout mutants of six of the 13 orphans display markedly reduced swarming capabilities in semisolid agar (Figure 4G), while 11 out of 13 mutants were significantly impaired for biofilm formation in vitro as compared with a wild-type control (Figure 4H). Taken together, these observations strongly implicate this set of orphans in the formation and/or proper function of fimbriae.

Several other prominent modules are shown in Figure 4I. These comprise the orphans YdiN, YdiL, and YdiM predicted (based on operon rearrangements) to functionally interact with several members of the Aro operon known to participate in the metabolism of shikimate, a precursor of aromatic amino acids. Consistent with this, *ydiN*, *aroD*, and *ydiB* are reportedly overexpressed when E. coli is grown in media containing shikimate as the sole carbon source [68]. Moreover, we found that deletion of either *ydiN* or *ydiB* resulted in defective metabolism of shikimate causing phenotypic auxotrophy for aromatic amino acids (Figures 4J and S3) as is observed for mutants of known aromatic amino acid biosynthetic genes (e.g., *aroA* and *aroD*) [69].

Other functional modules include *frlA*/*frlB*, part of the Frl operon of E. coli responsible for the import and metabolism of the alternative carbon source fructoselysine, together with the orphan YifK, which has sequence characteristics of a transporter [38], implicating it in electrochemical potential-driven uptake of this sugar. Conversely, two orphans, YecC and YecS, had functional associations consistent with linkages to amino acid and nucleotide metabolism, four (YagU, YqeG, YhaO, and YhaM) were linked to a putative module involved in transport and metabolism of threonine and serine, whereas three others (YjjI, YeiM, and YjjJ) were found in a module enriched for factors involved in nucleotide transport and degradation of deoxyribonucleosides.

Taken as a whole, these results suggest discrete functional relationships for many previously unannotated proteins, implicating certain orphans within specific pathways.

### Improved Functional Inference within an Integrated Network Framework

Examination of the extent of overlap between our physical and functional networks, both in terms of common binary interactions and shared components among the derived complexes (from PI) and modules (from GC), indicated that they are largely complementary (Table S11). Since a similar trend was also evident comparing other existing curated E. coli PI datasets (derived from either low-throughput or other high-throughput studies) with independent GC inferences (e.g., from STRING; Table S11), this presumably stems in part from the incomplete coverage obtained by these different approaches. Regardless, these observations imply that the union of PI and GC networks is necessary to capture the widest spectrum of biologically relevant interactions. Indeed, it has been shown previously that the combination of PI with functional genomic inferences, each statistically weighted according to dataset quality, can markedly improve both functional coverage and accuracy [59,61,70–72]. We therefore merged our experimental and predicted associations with the same method used to generate the unified GC network (Figure 2C; see Materials and Methods and Protocol S6 for details).

The resulting combined probabilistic network consisted of 80,370 high-confidence (probability ≥75%) putative pairwise interactions encompassing virtually the entire proteome of E. coli, including 2,769 (99%) annotated proteins and 1,375 (96%) functional orphans (Table S12). Graph analysis of this final integrated network (Protocol S7 and Table S13) indicated that the orphans tended to have a lower overall connectivity and betweenness centrality, measured as the number of shortest paths going through a given node, relative to annotated components, suggesting more peripheral positions in the integrated networks. However, the orphans also exhibited lower average closeness, defined as the average length of shortest paths between any two nodes, and had similar overall clustering coefficients, indicating that, in general, the orphans are functionally connected to, rather than isolated from, the annotated gene products. These observations implied that consideration of both the individual associations and overall placement of the orphans within the integrated interaction network would facilitate functional deduction.

We therefore devised a new network-based function prediction method (termed StepPLR; see Figure 2C and Materials and Methods) to exploit the global topological similarity among all the protein pairs and their corresponding functional annotations in the integrated network. Our method assigns functions to orphans based on the functional information from their first-order (direct) and second-order (indirect) annotated neighbors in the integrated network using penalized logistic regression models and a stepwise variable selection procedure to deduce optimal functional profiles (see Protocols S8 and S9 for details). We based our classifications on the discrete COG functional categories and on the hierarchical, multifunctional terms of the Gene Ontology (GO) [73,74] and MultiFun classification schemas [25]. To avoid potential sources of false predictions, we removed any proteins labeled with the evidence codes IPI (for inferred from protein interaction) and IGC (for inferred from genomic context method) when generating the GO reference set, as well as proteins in poorly characterized categories in COGs and MultiFun (Protocol S9 and Table S14).

As shown in Figure S4, StepPLR had better precision and recall prediction performance than several other widely used guilt-by-association procedures tested, such as majority-counting and chi-square–based methods (see Protocol S10 for details). Although the performance achieved for the different functional categories varied, our approach generated area-under-the-curve (AUC) values of 0.8 or higher for most of the COG (83%), GO (67%), and MultiFun (53%) categories (Table S15), and was relatively insensitive to the number of annotated proteins per function. Moreover, since our method exploited the correlation among the different categories, most orphans had multiple biologically consistent predicted functions (Table S16).

### Functional Neighborhoods

**Figure 5:**
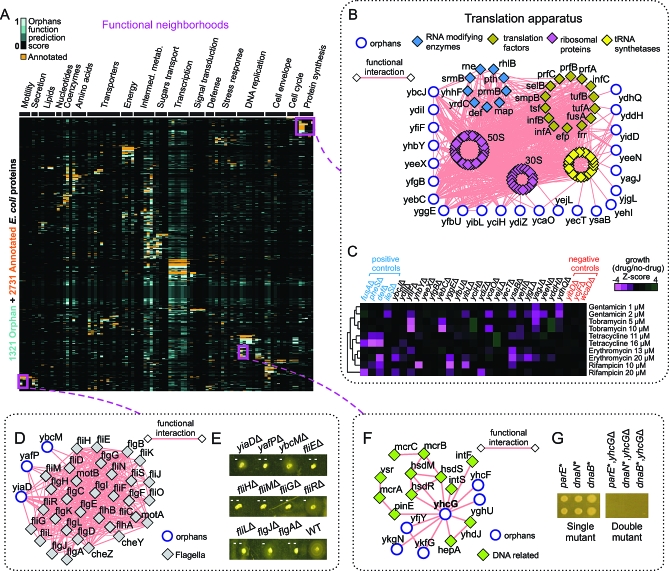
The Functional Neighborhoods of E. coli (A) A “clustergram” displaying existing annotations (orange) and the orphan predicted functions (this study; blue) for all the protein-coding genes of E. coli (*y*-axis) and their associated biological processes (*x*-axis) (complete terms are provided in Figure S5 and Table S16). Proteins were clustered using MCL based on the paired similarity of the functional annotations and predictions in this matrix to delimit “functional neighborhoods” (see Table S17 for listing). (B) Putative functional neighborhood showing high-confidence integrated functional interactions (combined PI and GC networks) of select orphans with the protein synthesis machinery. For clarity, individual names of ribosomal proteins and tRNA synthetases are not shown. (C) Heat map showing the differential sensitivity of orphan deletion strains to antibiotics targeting protein synthesis relative to the colony size in the absence of drug (see Protocol S11 for details). Mutants deleted for annotated proteins from this neighborhood are shown as positive controls, whereas deletion mutants lacking genes not contained within this neighborhood are shown as negative controls. (D) Neighborhood with three orphans putatively involved in flagellum assembly and motility. (E) Deletions of the corresponding components reduce swarming capability; single dash (-), moderately impaired motility; double dash (- -), strong repression. (F) Subnetwork of orphans associated with DNA enzymes. (G) Deletion of the orphan *yhcG* results in synthetic lethality when combined with hypomorphic alleles (as indicated by an asterisk [*]) of three essential DNA replication factors (*parE*, *dnaN*, and *dnaB*).

As displayed graphically in Figure 5A, our prediction procedure ultimately linked many of the orphans to specific, functionally related protein “neighborhoods”. We again made use of the MCL algorithm to objectively delimit functionally highly homogeneous (*p* < 0.0001) protein groupings based on the profile similarity of annotations and predictions (see Protocol S4 for details and Table S17 for listing). One notable example is the protein translation machinery (Figure 5B), which has 23 associated orphans. To independently verify the functional relevance of these assignments, we examined the effects of deleting the corresponding genes in terms of conferring sensitivity to drugs that inhibit protein synthesis. Consistent with expectation of a direct role in protein synthesis, and similar to loss of bona fide annotated translation factors and tRNA synthetases, the mutant strains exhibited statistically significant (*p* < 0.05) differential sensitivity as compared to wild type and unrelated gene mutants to a variety of antibiotics that selectively block protein translation (Figure 5C and Table S18).

We also examined an alternate group of orphans (YafP, YiaD, and YbcM) associated with the flagellar biogenesis and motility apparatus (Figure 5D). Single-gene knockout mutants of annotated components in this neighborhood exhibit decreased motility in semisolid agar as compared to wild-type E. coli strains [13]. Consistent with our functional predictions, we likewise found that deletion of *yafP* ablated cell motility in vitro (Figure 5E), similar to mutants lacking core flagellum motor encoding genes (e.g. *fliH* and *fliM*), whereas loss of *yiaD* and *ybcM* reduced swarming (i.e., decreased halo formation) to an extent comparable to perturbation of other established flagellar components (e.g., *flgJ* and *fliR*). Additionally, a previous study [75] using phenotypic complementation analysis had suggested that a *ybcM* ortholog in Yersinia enterocolitica is likely an AraC-type regulatory protein involved in controlling bacterial motility. Taken together, these results suggest that, akin to several other recently discovered novel motility components [13,76], these orphans are required for the proper assembly and/or subsequent locomotion of the E. coli flagella, a fundamental bacterial structure.

Many other orphans were predicted to have roles in other conserved biological systems, such as DNA replication. For example, as shown in Figure 5F, we identified the orphan YhcG in association with DNA processing enzymes, including the restriction complexes HsdMRS and McrABC, the integrases IntF and IntS, and the recombinase PinE. YhcG has sequence characteristics of the PD-(D/E)XK superfamily of nucleases involved in DNA recombination and repair [77]. Consistent with these observations, we found that deletion of *yhcG* results in a synthetic-lethal phenotype (Figure 5G) when combined with hypomorphic alleles of the replicative primosome (*dnaB*), DNA polymerase III (*dnaN*), and DNA topoisomerase IV (*parE*), consistent with a direct role in DNA replication or the resolution of critical intermediates.

### Novel Components of Bacterial Cell Envelope Biogenesis Pathways

**Figure 6:**
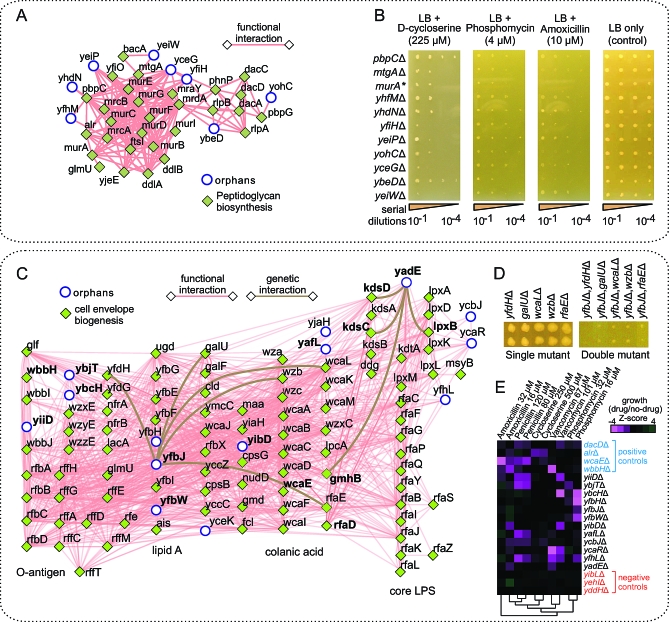
Functional Neighborhoods Involved in Cell Envelope Biogenesis (A) Functional neighborhood showing high-confidence integrated functional interactions (combined PI and GC networks) between components of the complete peptidoglycan biogenesis pathway and putatively functionally related orphans. (B) Serial-dilution assay showing perturbed growth of E. coli strains deleted for putative cell wall–related components (from [A]) in the presence of antibiotic inhibitors of cell wall assembly; an asterisk (*) indicates a hypomorphic allele of the essential gene *murA*. (C) Functional neighborhood of metabolic pathways involved in biogenesis of the bacterial cell envelope. Vertical groupings correspond to known pathways, whereas orphan components are tentatively positioned according to their interaction patterns; brown lines indicate aggravating genetic interactions recorded between the orphan *yfbJ* and *yadE* and annotated pathway components. (D) Conjugation-based double-mutant growth assays (only test results for *yfbJ* are shown). (E) Heat map showing the differential sensitivity of strains deleted for the orphans shown in (C) to antibiotics targeting the cell envelope. Mutants deleted of relevant annotated proteins are shown as positive controls, while mutants lacking genes not contained within this neighborhood are shown as negative controls.

Our functional predictions were particularly revealing about bacterial cell envelope biology, with implications for infectious disease and antibiotic susceptibility. Like other free-living microbes, E. coli is encased in a membranous cell envelope composed of proteins, lipids, and carbohydrates that serves as the interface to its environment and mammalian host, yet over a third of the approximately 1,000 predicted membrane-associated and periplasmic proteins of E. coli are presently functionally unannotated [38]. Figure 6A shows a set of eight orphans linked to a functional neighborhood along with 29 annotated proteins with established roles in the biogenesis of peptidoglycan, a major structural component of the bacterial cell wall that is a target of many antimicrobials. Consistent with our functional predictions, E. coli cells deleted for these same orphans exhibited differential sensitivity to various antibiotics that inhibit peptidoglycan assembly (Figure 6B). Moreover, the observed phenotypes were also characteristic of enzymes acting in the initial cytoplasmic and inner membrane stages of peptidoglycan biosynthesis, like *murA* and *mtgA*, rather than later periplasmic steps, like *pbpC*. This suggests orphans involvement in an early step in cell wall formation.

Figure 6C shows a second functionally homogeneous neighborhood composed of 14 orphans and 91 annotated proteins associated with pathways linked to the biosynthesis of lipopolysaccharide (LPS) and other core cell envelope components. Consistent with these assignments, subcellular protein localization studies based on green-fluorescent reporter fusions [78] have previously established that at least five of these orphans (YfbH, YfbJ, YfbW, YbjT, and YjdD) are physically tethered to the E. coli inner membrane, whereas signal peptides potentially mediating export across the inner membrane have been predicted for YafL and YadE [79]. Further, the interactions of annotated and orphan components within this neighborhood suggest specific relationships consistent with particular biological pathways. For instance, YfbJ, YfbH, and YfbW interact with Ugd and four other annotated proteins that participate in maturation of the lipid A anchor of LPS. Previous work [80] had suggested that an unknown transporter(s) shuttles lipid A intermediates between the cytoplasm and periplasm. Coincidentally, YfbJ and YfbW are paralogs that belong to a superfamily of multidrug efflux transporters [29], in which the C-terminus of YfbJ is cytoplasmic and that of YfbW periplasmic [78]. Since knockouts of the corresponding genes were recently reported to impair trafficking of lipid A precursors [81], YfbJ and YfbW appear to be the relevant transporters. Likewise, YadE and three other orphans (YjaH, YcaR, and YfhL) are predicted to function in Rfa-, Lpx-, and Kds-based pathways participating in KDO_2_-lipid A (i.e., core LPS) biosynthesis. Consistent with these predictions, we found that deletion of either *yfbJ* or *yadE* resulted in aggravating synthetic genetic interactions when combined with mutant alleles of genes in parallel pathways (Figures 6C and 6D).

Other orphans in this neighborhood (e.g., YiiD, YbjT, and YbcH) are predicted to interact either with the Rff and Rfb pathways involved in the generation of other important cell envelope constituents, such as enterobacterial common antigen and O-antigen. Likewise, YibD and YafL interact with Wca and Wz proteins that participate in biosynthesis of colanic acid (M-antigen). Moreover, although they are not formally classified within this route, we detected functional interactions of RfaD with LpxD, KdsA, and KdsC involved in the synthesis of ADP-l-glycero-d-*manno*-heptose, which is used by many enzymes for LPS production.

Similar to mutants deficient for enzymes annotated to participate in the cell envelope biogenesis, we found that deletions of the orphans in this neighborhood significantly (*p* < 0.05; see Protocol S11) perturbed cell viability upon exposure to antibiotics that block formation of the bacterial cell envelope (Figure 6E), substantiating our functional predictions.

### Functional Interactions of Orphans Extend beyond Proteobacteria

**Figure 7:**
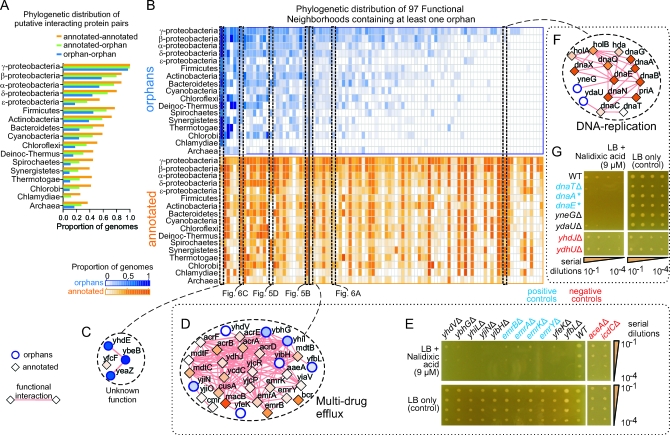
Evolutionary Conservation of Orphan Protein Function (A) Evolutionary conservation of orphan and annotated E. coli protein interaction partners in the integrated PI-GC network based on the co-occurrence of putative orthologs across fully sequenced prokaryotic genomes. (B) Phylogenetic distribution of the components of 97 functional neighborhoods with at least one orphan; the proportion of genomes showing conservation is indicated. (C) Atypical neighborhood illustrating broader conservation of orphans than annotated components; node shading reflects average phylogenetic conservation. (D) Representative neighborhood involved in drug efflux exhibiting similar phylogenetic distributions among its orphan and annotated components; analogous neighborhoods shown in preceding figures are indicated below [B]. (E) Serial-dilution assay showing the drug hypersensitivity of deletion mutants of the orphans listed in [D]. (F) DNA-replication neighborhood exhibiting a tendency of annotated components to be more widely distributed than the orphans. (G) Serial-dilution assay showing the perturbed growth of strains deleted for the two orphan components shown in [F] in the presence of an antibiotic inhibitor of DNA-replication; an asterisk (*) indicates hypomorphic alleles. Mutants deleted of relevant annotated proteins are shown as positive controls, while mutants lacking genes not contained within this neighborhood are shown as negative controls. LB, Luria Bertani medium.

To investigate the evolutionary significance of the putative functional associations detected in E. coli, we examined the presence of orthologs of each of the interacting orphan and annotated protein pairs among currently available prokaryotic genomes (see Materials and Methods and Protocol S12 for details). As might be expected from the more limited evolutionary scope of the orphans (cf. Figure 1G), functional interactions involving orphans were typically less broadly distributed than those of annotated proteins (Figure 7A), with the least-frequent distribution category consisting of interactions between the orphans themselves. Nevertheless, our analysis indicated extensive conservation of orphan associations across all sequenced forms of prokaryotic taxa. For instance, 5,553 putative interactions between orphan and annotated proteins, and 603 among orphan pairs alone, were predicted to be distributed as far as Archaea, again supporting the importance of orphans beyond that anticipated from the biases evident in previous functional characterizations of E. coli. Moreover, although it might be expected that GC predictions involving gene-order conservation and phylogenetic profile similarity would be biased towards highly conserved genes, both the PI (independent of gene conservation) and functional interactions (with only operon rearrangement–based predictions less dependent on gene conservation) follow the same tendencies. Hence, if there was residual bias towards finding functional interactions for only the most highly conserved genes, it is not pronounced.

We next compared the phylogenetic distributions of the orphan and annotated components of the 97 predicted functional neighborhoods with at least one orphan (Figure 7B). Our results indicate that only four neighborhoods have orphans that are more widely distributed than annotated members. An example, consisting of three highly conserved orphans linked to metabolism and one poorly conserved annotated component is shown in Figure 7C. This pattern suggests the orphans are of primary functional significance. In contrast, most (57) other neighborhoods, including several described in previous sections (cf. Figures 5B, 5D, 6A, and 6C), generally have similar distribution profiles of orphan and annotated members (Pearson correlation >0.5), suggesting equal participation across diverse biological processes despite the incomplete previous classifications. For example, the average component distributions of one such representative neighborhood involved in drug efflux is shown in Figure 7D. Six of the seven orphans of this particular neighborhood are predicted to localize to the E. coli inner membrane [38]. We found that deletion of these orphans results in hypersensitivity to an otherwise exported drug, similar to that observed upon loss of the annotated components (Figure 7E), suggesting equal participation in the maintenance of cell homeostasis.

Conversely, annotated components exhibited a broader phylogenetic distribution in the remaining 36 neighborhoods. For example, two orphans (YneG and YdaU) linked to DNA replication (Figure 7F) were far more evolutionarily restricted than their annotated counterparts. Nevertheless, deletion of these same genes markedly reduced cell viability upon exposure to an inhibitor of DNA replication (Figure 7G) similar to hypomorphic strains of their broadly distributed replication partners DnaT, DnaA, and DnaE. Therefore, the sparser distribution of these orphans, and those of the other 35 neighborhoods, may reflect roles as critical accessory factors in their respective biological systems, perhaps to fine tune cellular responses to particular environmental adaptations and selective pressures (e.g., exposure to antibiotics).

## Discussion

Defining the precise biological roles and relationships of bacterial gene products in an often dynamically changing physiological context is a challenging proposition. Historically, systematic assessments of protein function in bacteria have tended to rely on molecular inferences based on sequence alignments and domain architectures, whereas experimental characterization has traditionally been driven by specific scientific interests rather than with the aim of providing the broader community with unbiased collections of functionally related proteins and phenotypes. Since the biological role of a protein is not necessarily reflected in its primary sequence, the elucidation of molecular interaction networks can provide an alternate perspective even in the absence of detailed phenotypic data [16,71]. Here, we have opted to view a model microbial cell mechanistically as a series of modular molecular interaction networks that underlie the major biochemical processes that mediate cell homeostasis and proliferation, wherein the functional attributes of particular gene products are reflected in their overall patterns of associations.

To this end, we have generated an extensive compendium of physical and functional linkages covering almost the entire protein-coding complement of E. coli. This led to the elucidation of hundreds of putative soluble multiprotein complexes and functional modules encompassing virtually all the many gene products currently lacking public annotations. Although existing integrative probabilistic interaction databases such as STRING [61] and EcID [82] provide valuable additional binary interactions that are potentially useful for protein function prediction or as complementary evidence to those reported in this study, our machine learning strategy goes beyond describing binary interactions by explicitly describing the most probable biological functions of the orphans. Of particular noteworthiness, our functional predictions and phylogenetic projections associate a sizeable fraction of the functional orphans with core bacterial processes, suggesting they may have previously eluded detection in part due to prior analytical biases.

Since the various methods used in this study to discover different types of molecular relationships also have their own intrinsic biases, complementary information was obtained through data integration. The limited overlap between the high-confidence physical and functional interaction networks presumably stems in part from the incomplete coverage typically achieved by high-throughput experiments and their methodological differences [13,83]. For example, certain orphans were difficult to evaluate by GC methods due to a lack of apparent orthologs at medium-to-high evolutionary distances, which hinders comparative genomic inferences. Likewise, although we performed large-scale tandem affinity tagging and purification under near-native physiological conditions to generate highly purified preparations of stable, endogenous multiprotein complexes, we did not achieve complete coverage of the proteome. We did not attempt to purify a large number of membrane-associated proteins, which require specialized solubilization procedures, whereas the soluble proteins that we failed to tag or detect by mass spectrometry were presumably either of very low abundance or not expressed in our growth conditions.

Comparison of our physical interaction network with analogous public datasets produced for other model species, such as worm, fly, yeast, and even the bacterium Helicobacter pylori, revealed very limited (<1%) overlap. These observations are congruent with recent findings by Rajagopala and colleagues [13] showing that only a third (49) of the 173 experimentally derived PI in the cell motility network of the spirochete Treponema pallidum predicted to occur in the ɛ-proteobacteria Campylobacter jejuni on the basis of orthology could subsequently be confirmed by targeted two-hybrid testing. The limited overlap between proteomic datasets presumably reflects a combination of incomplete coverage by various experimental assays, methodological differences and evolutionary divergence.

The observation that the intersection of functional genomics inferences with low-throughput curated physical interaction data is somewhat higher (cf. Table S11) might be explained by two nonmutually exclusive ways: first, protein–protein interactions reported in the literature based on traditional biochemical methods might be biased towards the most evolutionarily conserved multiprotein complexes, which tend to be enriched for essential components with broadly distributed phylogenetic profiles that are more easily and accurately predicted by GC methods; second, the relatively high sensitivity of the two complementary forms of protein mass spectrometry used in this study may have resulted in the detection of lower-abundance orphan proteins that have previously not been studied in depth.

The last point is consistent with the notion that different proteomic methods capture different PI types [83]. Hence, alternative proteomic methods, such as two-hybrid screens [13,84–86] or in vivo protein-fragment complementation assays [87], may be better suited for detecting certain PI currently underrepresented in our dataset. In a similar vein, additional functional relationships will undoubtedly be uncovered by different experimental and computational procedures, such as high-throughput comparative analysis of mutant cellular phenotypes [2], genome-wide genetic interaction screens [88,89], and automated text mining [90,91].

The topological properties inherent to biological networks (e.g., their hierarchical organization and degree distributions) combined with incomplete interactome coverage make establishing definitive functional groupings difficult [92]. Our approach was to take into account both the correlations among functional categories and the overall topological structure of the integrated network to generate a more balanced probabilistic model. Whereas alternative methods may provide enhanced interpretations of the organizational properties of the PI and GC networks, the functional enrichment and experimental validations established here suggest that our network-based computational inferences provide a reasonable perspective for exploring bacterial protein function. Similar strategies have resulted in powerful predictors of protein function in Eukaryotes [49,72,93–95]. The potential tradeoff is that additional error or uncertainty may have occasionally been introduced by assuming functional similarity among more loosely connected proteins. Moreover, the probabilities associated with particular functional terms may not be directly comparable. Functional orphans associated with very well-characterized biological processes are more likely to be correctly assigned by computational methods [72], whereas those associated with relatively poorly studied pathways will tend to remain obscure. Nonetheless, they can be grouped together on the basis of specific PI, GC, or even other functional associations (cf. Figure 7C) and hence serve as functional groupings rather than isolated entities.

In general, the high-confidence functional relationships we inferred for E. coli could be validated by independent experimental tests, and can be extrapolated to other bacterial species, including pathogens. Over 35% of the orphans find orthologs as far away as Archaea, and hence are likely associated with the same basic housekeeping processes we predict for E. coli, such as formation of the cell wall and protein synthesis. For instance, we have established putative roles in sugar and lipid metabolic pathways for several dozen evolutionarily conserved orphans that appear to be critical for proper biogenesis of the bacterial cell envelope, and hence may represent novel targets for antibiotic development. Conversely, our systematic comparisons also revealed some unique aspects of the orphans in the evolutionary history of E. coli, such as the potential fimbriae factors that appear to be restricted to Enterobacteriaceae. One interpretation is that orphans with limited phylogenetic distributions contribute to fine tuning of adaptive physiological responses upon changing environmental conditions, as previously suggested for peripheral metabolic genes acquired by horizontal transfer [96]. Conversely, the fact that the interactions of orphans with annotated proteins show a higher proportion of conservation across taxa implies that conserved biological systems are still to be discovered, and whose member contributions could extend across evolutionary domains. The physical and functional associations reported here are therefore presented as a Web-accessible public resource called “eNet” (http://ecoli.med.utoronto.ca; see Protocol S13 for details) to facilitate exploration of the fundamental molecular biology of bacteria in general and for hypothesis-driven studies of unique aspects pertaining to E. coli more specifically.

## Materials and Methods

### PI network generation.

Large-scale SPA tagging and purifications were performed essentially as previously described [4,32]. Briefly, a DNA cassette encoding the SPA-tag and a selectable marker flanked by gene-specific targeting sequences was amplified by PCR using primers with homology to a selected locus. The cassette was then transformed and integrated using homologous recombination in the lysogenic E. coli strain DY330 (W3110 background), which harbors the highly efficient λ-phage–encoded homologous recombination enzymes *exo*, *bet*, and *gam* under the control of the temperature-sensitive CI857 repressor (the “Red” system), to create a C-terminal fusion with the protein of interest. Strains in which the PCR product was integrated were subjected to antibiotic selection, and tagged protein expression was confirmed by western blotting. Tagging primer sequences are available upon request.

Two complementary mass spectrometry techniques (gel-based MALDI peptide mass fingerprinting and gel-free LCMS shotgun sequencing) were used to detect physically interacting proteins. Details about the large-scale strain culture, protein extraction and purification, and protein identification procedures are provided in Protocol S3. Scoring of tentative PI from the LCMS and MALDI assays was conducted using a logistic regression model using reference PI obtained by low-throughput experiments curated in the DIP, BIND, and IntAct databases [35–37] as a positive training set. Our negative training set consisted of pairs of proteins in which one component was experimentally determined or predicted with high confidence to be cytoplasmic and the other residing in the outer membrane or the periplasm [38]; inner membrane proteins were discarded from this negative dataset since they are in physical proximity (and hence could potentially physically interact) to cytoplasmic and periplasmic proteins. Our logistic regression procedure also took into account the degree of consistency of copurifying protein pairs, balancing the tradeoff between “spoke” and “matrix” representation models of interactions within copurified groups of proteins to decrease the false discovery rate. We then combined the scores derived from LCMS and MALDI into a a single PI network using a previously established procedure for integrating probabilistic networks [61], which assumes the reliabilities of associations generated by these methods are independent (see Protocol S6 for details). To facilitate independent critical evaluation, all our processed interaction data is available through the Web site in HUPO-PSI molecular interaction reporting format (standard level 2.5).

### GC network generation.

The four GC methods used to predict functional interactions among E. coli proteins were based on: (1) functional linkages among genes which fuse to form a single open reading frame in at least one other genome, i.e., gene fusion [48]; (2) the mutual information of the coordinated presence or absence of pairs of genes across a set of 440 nonredundant genomes, i.e., phylogenetic profiles [51,97]; and the natural chromosomal association of bacterial genes in operons as detected by two alternative methods, namely (3) the tendency of genes forming operons to show small intergenic distances [98,99], and (4) the conservation of gene order, in which a confidence value for each pair of adjacent genes in the same strand was used as indicator that those genes likely form an operon, as compared with the conservation of adjacent genes in opposite strands [53]. For the last two methods, subsequent operon rearrangements were detected by genomic mapping of orthologs across 440 nonredundant bacterial genomes [55].

For all four GC methods, we used the BLAST-BDBHs as an operational definition of orthology (see Protocol S5 for details). To avoid circularity, the prediction scores of the four GC methods were benchmarked separately using proteins belonging to the same metabolic pathway according to EcoCyc [11] as positive reference set, and proteins in different pathways as negatives (Protocol S5). A single, unified high-confidence functional association network was then constructed by integrating the interaction predictions generated by the four GC methods using the same scoring model [61] used to integrate the MALDI and LCMS data (Protocol S6).

### Clustering of networks.

Protein clusters were generated from three different networks using MCL [40] (Figure 2): (1) the PI network (generating protein complexes); (2) the unified GC network (generating functional modules); and (3) the function prediction/annotation profiles derived from the integration of PI and GC networks (generating functional neighborhoods). The core idea of MCL is to simulate random walks (i.e., flux) among the proteins (nodes) within each network to delimit regions with high flux, taking into account the connectivity and weight of interaction edges. In this work, edge weights correspond to the likelihood of pairwise protein interactions in each network. In each case, the global MCL inflation parameter, which tunes the granularity of the delimited clusters, was optimized by balancing the mass fraction of clusters and efficiency of partitions (see Protocol S4 for details). The resulting clusters were individually assessed for functional homogeneity in terms of COG annotations as described previously [100] (Protocol S4).

### Network-based function prediction and benchmarking.

Our algorithm (StepPLR) for assigning biological functions is essentially a network topology–based method in which the functions of the orphans are predicted based on the functions of their associated annotated proteins in the immediate (direct) and adjacent (indirect) network vicinity (see Protocol S9 for details). Briefly, a single network integrating the high-confidence PI and GC probabilistic networks was first created using the same scoring model [61] used to integrate the PI data and the four GC networks. The weighted topological overlap [101] between each pair of protein nodes in the integrated network was then calculated to determine the correlated functional profiles based on a penalized logistic regression model (see Protocol S8 for details). Finally, a stepwise variable selection procedure to optimize function profiles in the final logistic regression was used (see Protocol S9 for details). Only functional categories with at least 15 annotated E. coli proteins were used in our integrated functional association network (see Table S14): 18 COG classes, corresponding to bacterial protein functions; 19 biological classes from MultiFun, in which the proteins can have multiple annotations based on different classification criteria; and 51 biological process classes in GO. Other guilt-by-association representative methods (e.g., majority-counting and chi-square–based) were also evaluated (results shown in Figure S4A). Expanded descriptions of benchmarking and other computational procedures of our function prediction algorithm are provided in Protocols S9 and S10.

### Experimental validation of functional predictions.

Orphans were selected for experimental validation of functional predictions based on the following criteria: (1) the orphan was predicted to perform a function for which a suitable phenotypic assay was previously reported (e.g., an antibiotic targeting the associated function was available); (2) the orphan was clearly grouped with select annotated genes, allowing the inclusion of positive as well as negative controls; and (3) the orphan had high (>0.8 confidence) function prediction score(s). Antibiotic susceptibility assays were performed by pinning orphan and annotated gene knockout mutants [2] onto solid media plates in the presence or absence of antibiotics, and then imaging and comparing colony sizes. Details of the antibiotic sensitivity, translation, and auxotrophy assays are provided in Protocol S11. Motility assays were performed with overnight E. coli strain cultures pinned onto rectangular Petri dishes (Singer) containing semisolid swarming agar (LB medium with 0.25% agar). The swarming phenotype was classified visually based on the cell spreading-halo diameter observed after approximately 8 h incubation at 32 °C. Biofilm formation assays were conducted essentially as described in [102], with replicate data normalized relative to wild-type controls (Protocol S11). Epistatic genetic interactions between pairs of gene mutants in E. coli were identified using a newly developed conjugation-based screening method [88]. Briefly, a drug resistance–marked query gene deletion in a high-frequency recombination donor strain was crossed into either single-gene deletion knockout mutants from the Keio strain collection [2] or select essential gene hypomorphs to generate double mutants. After double drug selection, synthetic lethal or sick phenotypes were scored visually according to measured colony sizes (Protocol S11).

## Supporting Information

Figure S1 Influence of Gene Expression and Subcellular Localization on PI Detection (37 KB PDF)

Figure S2 Functional Homogeneity, Connectivity, and Cluster Size in PI Complexes and GC Modules (38 KB PDF)

Figure S3 Auxotrophy of *ydiB*Δ and *ydiN*Δ for Shikimic- and Aromatic Amino Acids (620 KB PDF)

Figure S4 Precision and Recall Benchmark Analysis of Function Prediction Algorithms (29 KB PDF)

Figure S5 Clustered Annotation Terms and Functional Neighborhoods (348 KB PDF)

Protocol S1 Gene Product Attributes (25 KB DOC)

Protocol S2 Metagenomic Analysis (52 KB DOC)

Protocol S3 Proteomic Analysis (85 KB DOC)

Protocol S4 Clustering to Define Protein Complexes, Functional Modules, and Neighborhoods (34 KB DOC)

Protocol S5 Prediction of Functional Interactions by Genomic Context (72 KB DOC)

Protocol S6 Global Integration of Different Data Sources for Function Prediction (30 KB DOC)

Protocol S7 Analysis of Topological Network Properties (28 KB DOC)

Protocol S8 Calculating Node Similarity in the Integrated Functional Association Network (34 KB DOC)

Protocol S9 Network-Based Protein Function Prediction (154 KB DOC)

Protocol S10 Comparison of Our New Algorithm (StepPLR) with Established Methods (33 KB DOC)

Protocol S11 Experimental Validations of Functional Predictions (47 KB DOC)

Protocol S12 Analysis of Functional Interactions of Orphans Extend beyond Proteobacteria (22 KB DOC)

Protocol S13 Public Web Server Details (38 KB DOC)

Table S1 E. coli K-12 (W3110) Gene Annotations and Properties (1.73 MB XLS)

Table S2 Genomic and Metagenomic Conservation of E. coli K-12 (W3110) Genes (2.17 MB XLS)

Table S3 Performance Comparison of Different Methods in PI Analysis (8 KB PDF)

Table S4 Reciprocal PI (75 KB XLS)

Table S5 Promiscuous “Hub” Proteins Filtered from the PI Dataset (7 KB PDF)

Table S6 Integrated PI Network Data (2.15 MB XLS)

Table S7 Predicted Protein Complexes (40 KB TXT)

Table S8 Integrated Functional Interaction Data Generated by Genomic Context (4.08 MB TXT)

Table S9 Predicted Functional Modules (81 KB TXT)

Table S10 Association of the Orphan Genes in Fimbrial Module to Biofilm Gene Expression Studies (30 KB XLS)

Table S11 Comparison of PI versus GC Interactions (8 KB PDF)

Table S12 Integrated PI and GC Network (3.49 MB TXT)

Table S13 Topological PI, GC, and Integrated Network Properties (24 KB XLS)

Table S14 Gold Standards of Functional Categories Used for Function Prediction (1.15 MB XLS)

Table S15 Function Prediction Performance Measured by AUC Scores (35 KB XLS)

Table S16 Orphan Gene Properties (2.25 MB XLS)

Table S17 Neighborhood Members (78 KB TXT)

Table S18 Relative Fitness and Functional Enrichment Analyses of Orphan and Annotated Genes Using Drug Screens (151 KB XLS)

## Abbreviations

BIND: Biomolecular Interaction Network Database
CAI: codon adaptation index
COG: Clusters of Orthologous Group
DIP: Database of Interacting Proteins
GC: genomic context
GO: Gene Ontology
LCMS: liquid chromatography-tandem mass spectrometry
LPS: lipopolysaccharide
MALDI: matrix-assisted laser desorption/ionization mass spectrometry
MCL: Markov clustering algorithm
PI: physical interactions
STRING: Search Tool for the Retrieval of Interacting Genes/Proteins
