# Supplementary material for: Global Functional Atlas of Escherichia coli Encompassing Previously Uncharacterized Proteins
Source: PLoS Biol. 2009 Apr 28;7(4):e1000096. doi: 10.1371/journal.pbio.1000096 (PMC2672614; doi:10.1371/journal.pbio.1000096)
Supplement: Figure S1 — (37 KB PDF) [file pbio.1000096.sg001.pdf]

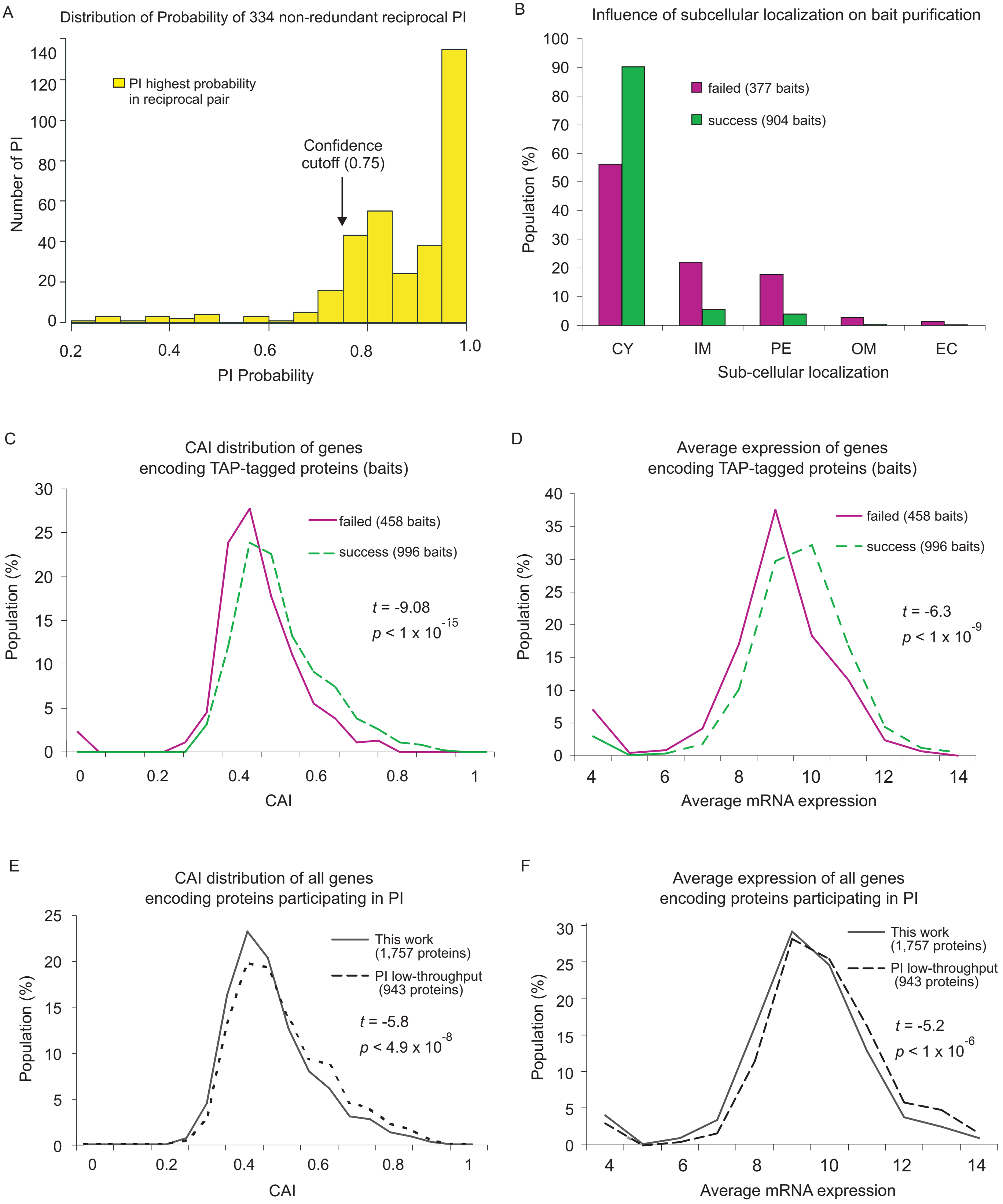

Figure S1 – Influence of gene expression and subcellular localization on PI detection.
(A) A total of 334 non-redundant reciprocal PI were recovered in this study. As most (71%) of these have a calculated probability score of 0.75 or greater in both directions (protein1→protein2 and protein2→protein1), we used this value as a final confidence cutoff. **(B to D)** One third (469) of the ~1500 proteins TAP-tagged in this study failed to be detected by both LCMS and MALDI. We consider both low expression and membrane-associated localization as potential causes for incomplete bait recovery failure since **(B)** ~35% of these of failed baits appear to be bound to the cell-envelope (Diaz-Mejia et al. 2009; CY: cytoplasmic; IM: inner-membrane; PE: periplasmic; OM: outer-membrane; EC: extra cellular), while **(C)** ~40% show evidence for a relatively low expression level, estimated by both low (< 0.7) CAI and **(D)** low (< 0.7) average mRNA expression levels (Faith et al. 2007). Conversely, in general proteins involved in PI reported in low-throughput studies (deposited in DIP, BIND and IntAct) are more highly expressed, and presumably more abundant, than those analyzed in this study, judging from their higher relative distributions in terms of both **(E)** CAI and **(F)** average mRNA expression.
