# Supplementary material for: Global Functional Atlas of Escherichia coli Encompassing Previously Uncharacterized Proteins
Source: PLoS Biol. 2009 Apr 28;7(4):e1000096. doi: 10.1371/journal.pbio.1000096 (PMC2672614; doi:10.1371/journal.pbio.1000096)
Supplement: Figure S2 — (38 KB PDF) [file pbio.1000096.sg002.pdf]

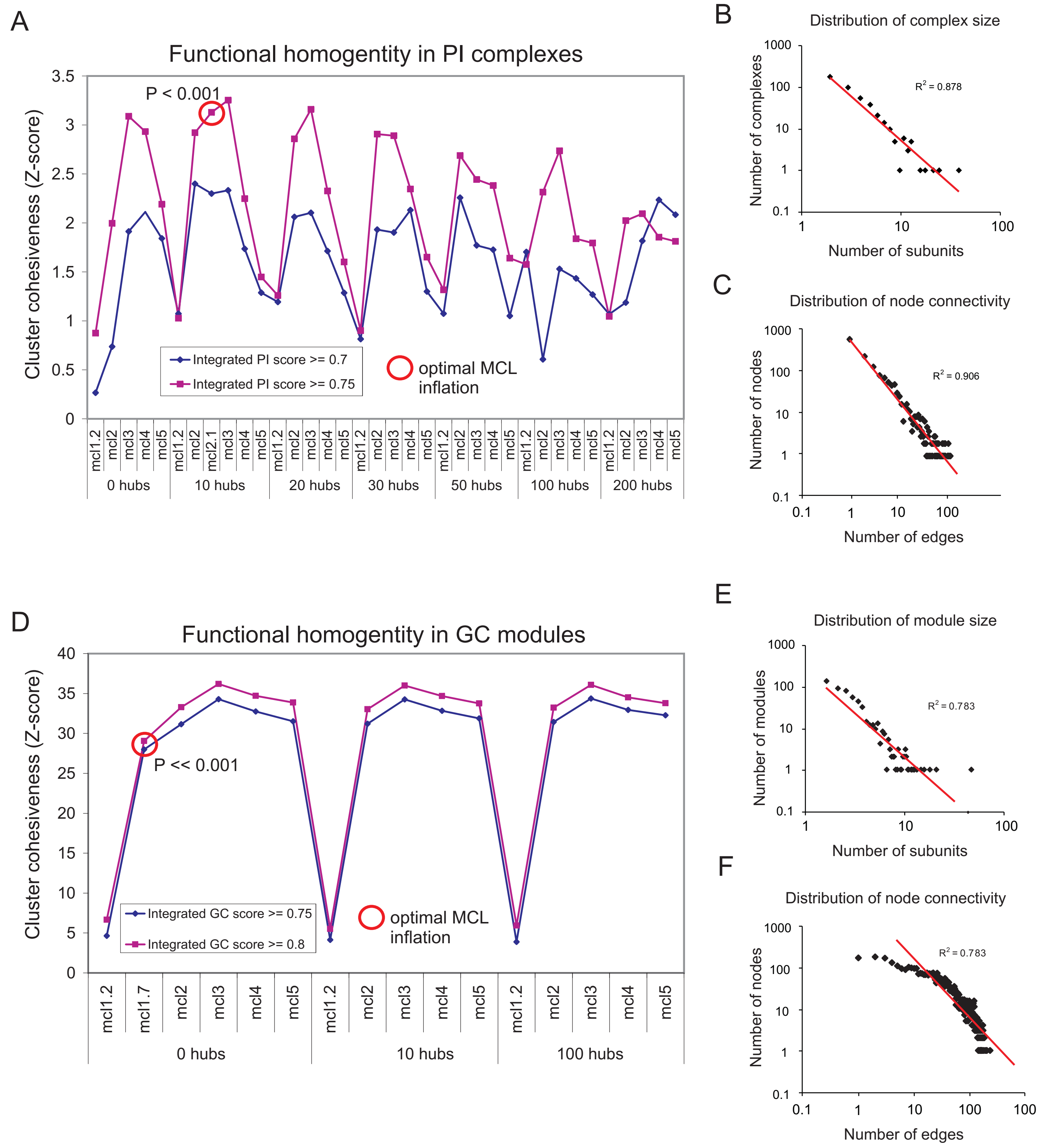

Figure S2 – Functional homogeneity, connectivity and cluster size in PI complexes and GC modules.
Three parameters were tuned to determine the optimal structural and functional partitioning of networks by MCL into PI multiprotein complexes and GC functional modules: namely, the MCL inflation value, the interaction scores, and the number of highly-connected hubs removed from consideration from each network. Optimization of these parameters resulted in a significantly greater functional homogeneity as compared with null models for both the PI and GC networks, as shown in panels **(A)** and **(D)**, respectively. The distribution of members in the resulting final optimized sets of **(B)** putative complexes and **(E)** functional modules fits a power-law distribution. Conversely, while the degree of connectivity of the high-confidence PI network **(C)** follows a typical power-law distribution, the degree of connectivity of the GC network **(F)** shows an increased number of nodes with 10 to 100 interactions, deviating from a power law distribution.
