# Supplementary material for: Global Functional Atlas of Escherichia coli Encompassing Previously Uncharacterized Proteins
Source: PLoS Biol. 2009 Apr 28;7(4):e1000096. doi: 10.1371/journal.pbio.1000096 (PMC2672614; doi:10.1371/journal.pbio.1000096)
Supplement: Figure S3 — (620 KB PDF) [file pbio.1000096.sg003.pdf]

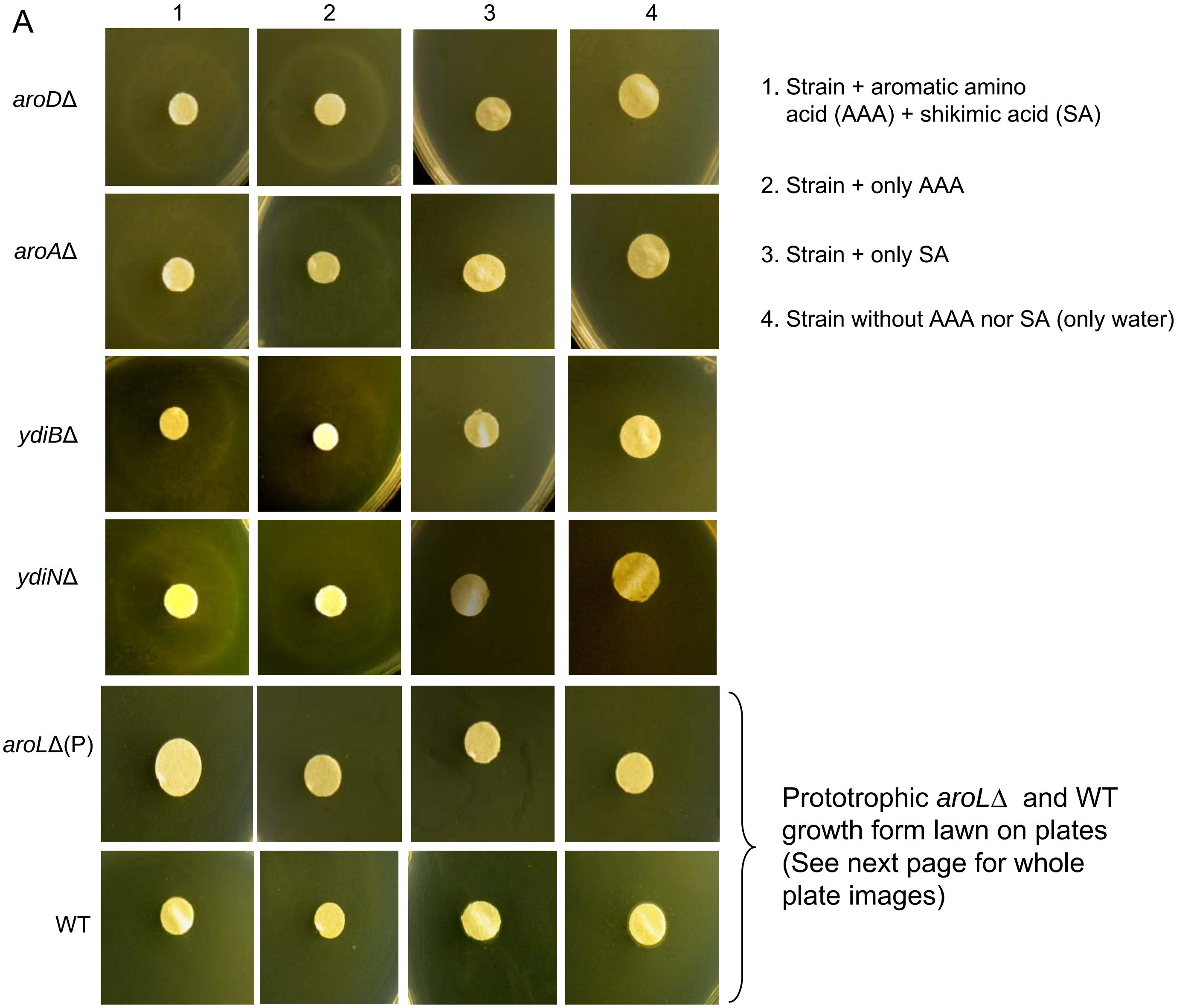

Figure S3 – Auxotrophy of of *ydiB*Δ and *ydiN*Δ for shikimatic- and aromatic amino acids.
(A) Images showing the shikimatic-acid and aromatic amino acid auxotrophy of ‘knock out’ mutants deleted for orphan genes (*ydiB* and *ydiN*) evident by defective growth on limited minimal media agar, similarly to mutations in genes (*aroD* and *aroA*) known to participate in the biosynthesis of these compounds. The *aroL* prototrophic (P) mutant and wild type BW25113 strains are specificity controls. Growth of these last two strains was observed in the whole dish (bottom pictures). The concentration of shikimic acid dipped to paper discs was 400 µg/mL, while the concentration of each compound added in the aromatic amino acid mixture was as follows: 1mM phenylalanine, 1mM tyrosine, 0.5mM tryptophan, 0.1mM *p*-aminobenzoic acid, and 0.4mM *p*-hydroxybenzoic acid. See Protocol S11 for details. **(B) See next page for** *aroL*Δ(P) and WT whole plate images.

B

*aroL*Δ(P)

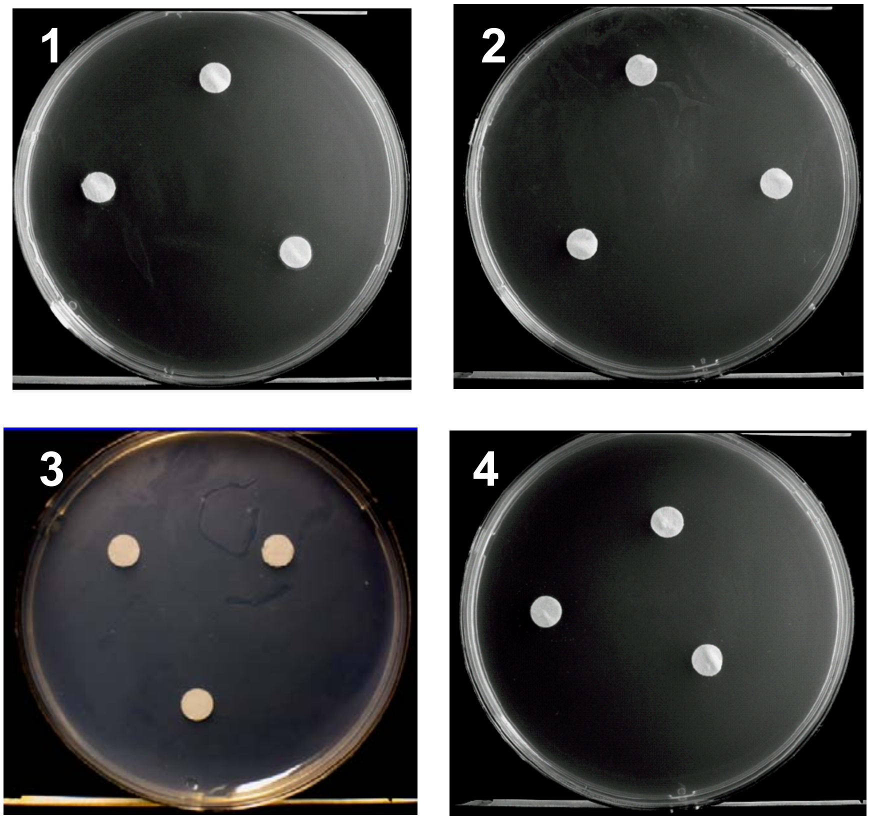

1. Mutant + AAA + SA
2. Mutant + only AAA
3. Mutant + only SA
4. Mutant without AAA nor SA (only water)

WT

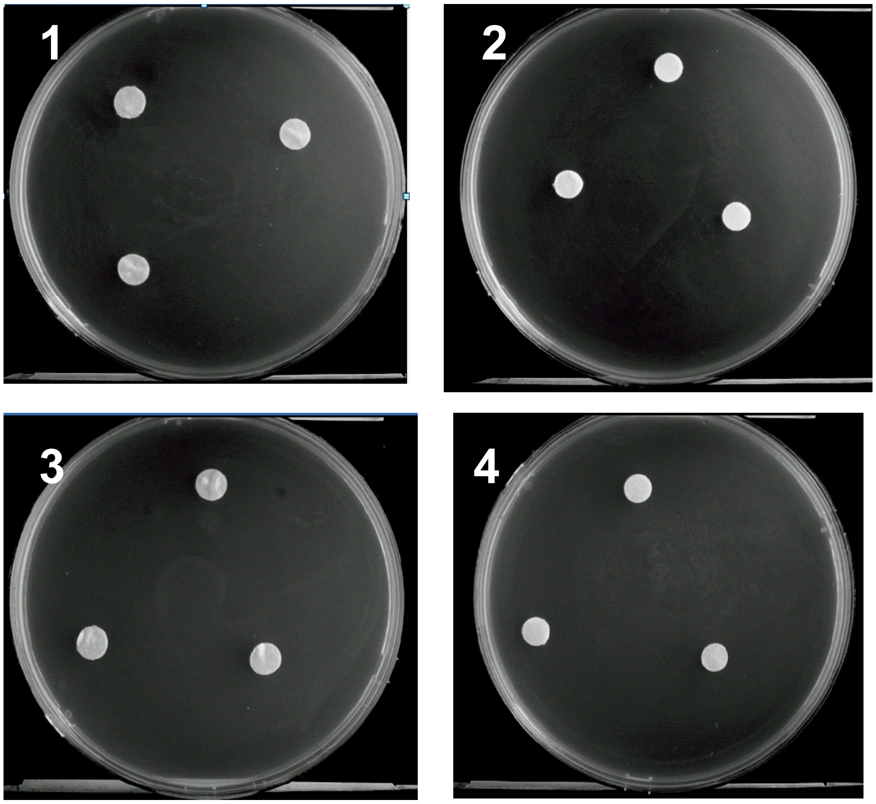

1. WT + AAA + SA
2. WT + only AAA
3. WT + only SA
4. WT without AAA nor SA (only water)
