# Supplementary material for: Global Functional Atlas of Escherichia coli Encompassing Previously Uncharacterized Proteins
Source: PLoS Biol. 2009 Apr 28;7(4):e1000096. doi: 10.1371/journal.pbio.1000096 (PMC2672614; doi:10.1371/journal.pbio.1000096)
Supplement: Figure S4 — (29 KB PDF) [file pbio.1000096.sg004.pdf]

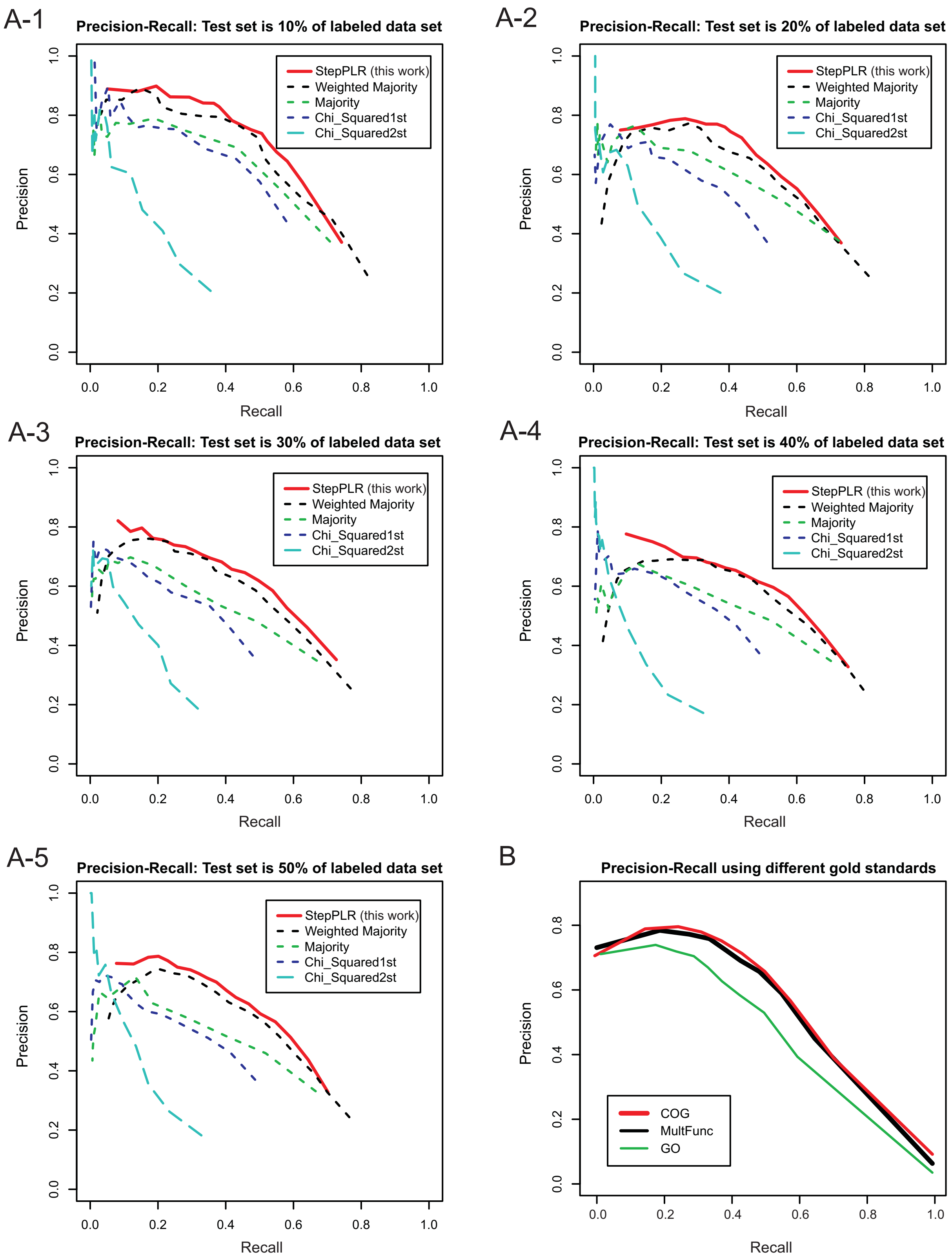

FigureS4 – Precision and recall benchmark analysis of function prediction algorithms.
(A-1 to A-5) Figures showing comparisons of the precision vs. recall performance for the predictions made by our new function prediction algorithm (StepPLR) along with those of four alternate existing (published) methods (belonging to two types: majority-counting and chi-squared) based on a set of 'gold-standard' functional categories from the MultiFun classification schema using 10%, 20%, 30%, 40% and 50% of labeled data as test data. **(B)** Figure showing the precision vs. recall of the cross-validation predictions generated by StepPLR against a set of 'gold-standard' functional categories from COG, MultiFun or GO.
