# Supplementary material for: Global Functional Atlas of Escherichia coli Encompassing Previously Uncharacterized Proteins
Source: PLoS Biol. 2009 Apr 28;7(4):e1000096. doi: 10.1371/journal.pbio.1000096 (PMC2672614; doi:10.1371/journal.pbio.1000096)
Supplement: Figure S5 — (348 KB PDF) [file pbio.1000096.sg005.pdf]

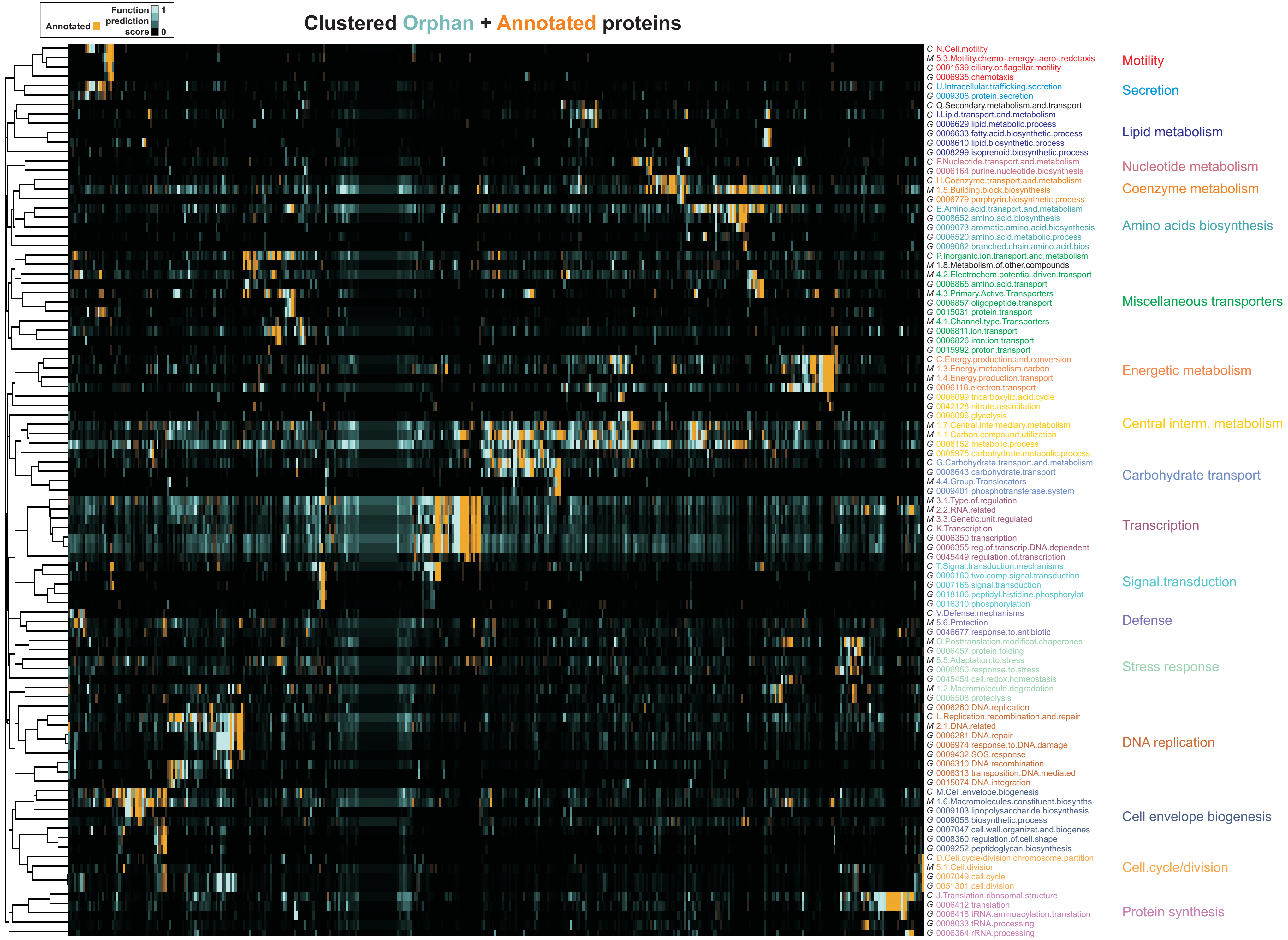

FigureS5 – Clustered annotation terms and functional neighborhoods.
Complete dendogram showing the functional relatedness and listing of the functional categories (y-axis) associated with the 2D 'clustergram' shown in Figure 5A based on the functional predictions and existing annotations for all the orphan and annotated proteins of *E. coli*. The single letter code (Italic font) shown before each functional term indicates COG (C), MultiFun (M) and GO (G) derived categories, respectively.
