# Supplementary material for: Global Functional Atlas of Escherichia coli Encompassing Previously Uncharacterized Proteins
Source: PLoS Biol. 2009 Apr 28;7(4):e1000096. doi: 10.1371/journal.pbio.1000096 (PMC2672614; doi:10.1371/journal.pbio.1000096)
Supplement: Protocol S1 — (25 KB DOC) [file pbio.1000096.sd001.doc]

**Protocol S1 – Gene product attributes**

Curated biomedical citations were obtained from UniProt as downloadable (*.dat) annotation files, removing genome sequence alone references and uninformative descriptions like ‘predicted’, ‘hypothetical’, or simply ‘conserved’ proteins. We defined as functional orphans (i.e. genes of unknown function) those genes that meet all the three following criteria: *i*) The genes have a name derived from the systematic "y-name" nomenclature [1], which is indicative of an unknown gene; *ii*) The genes do not belong to an EcoCyc known pathway; *iii*) The genes do not have a proper functional description in the GenProtEC knowledgebase system (a non-proper description would be "predicted", "hypothetical" and "conserved protein").

Molecular weights were calculated using the *pepstats* program of the EMBOSS suite [2], version 5.0.0. Codon Adaptation Index (CAI) was calculated by finding the genes coding for ribosomal proteins in *E. coli* K-12, building a reference (ribosomal) codon usage table with the EMBOSS program *cusp*; and finally calculating the CAI for all of the annotated coding genes of *E. coli* K-12 against the ribosomal-protein codon usage table with the EMBOSS program *cai*. Statistical analyses on the differences between functional orphans and functionally characterized genes were performed using the R statistical package [3].

**References**

1. Rudd KE (1998) Linkage map of Escherichia coli K-12, edition 10: the physical map. Microbiol Mol Biol Rev 62: 985-1019.

2. Rice P, Longden I, Bleasby A (2000) EMBOSS: the European Molecular Biology Open Software Suite. Trends Genet 16: 276-277.

3. R_Development_Core_Team (2006) R: A language and environment for statistical computing. Vienna, Austria: R Foundation for Statistical Computing.
