# Supplementary material for: Global Functional Atlas of Escherichia coli Encompassing Previously Uncharacterized Proteins
Source: PLoS Biol. 2009 Apr 28;7(4):e1000096. doi: 10.1371/journal.pbio.1000096 (PMC2672614; doi:10.1371/journal.pbio.1000096)
Supplement: Protocol S2 — (52 KB DOC) [file pbio.1000096.sd002.doc]

**Protocol S2 – Metagenomic analysis**

Metagenomic BLASTP comparisons were performed against annotated microbiomes available at the DOE Joint Genome Institute [1] (<http://img.jgi.doe.gov/cgi-bin/m/main.cgi>): AMO, Methane Oxidizing Archaea [2]; Acid Mine Drainage [3]; anaerobic ammonium oxidation (anammox) Bacteria [4]; human gut communities [5]; Mouse gut communities [6]; Sludge [7,8]; and microbial communities from whale carcasses (whalefall) [8]; plus the non-redundant environmental sequences available at NCBI as the "nr_env" BLAST database (<ftp://ftp.ncbi.nih.gov/blast/db/>) as of July 2007, consisting mostly of the enormous amount of data from the marine metagenomic sequences of the Sorcerer II Ocean Sampling expedition [9] and the Sargasso sea survey [10].

**References**

1. Markowitz VM, Ivanova N, Palaniappan K, Szeto E, Korzeniewski F, et al. (2006) An experimental metagenome data management and analysis system. Bioinformatics 22: e359-367.

2. Hallam SJ, Putnam N, Preston CM, Detter JC, Rokhsar D, et al. (2004) Reverse methanogenesis: testing the hypothesis with environmental genomics. Science 305: 1457-1462.

3. Tyson GW, Chapman J, Hugenholtz P, Allen EE, Ram RJ, et al. (2004) Community structure and metabolism through reconstruction of microbial genomes from the environment. Nature 428: 37-43.

4. Strous M, Pelletier E, Mangenot S, Rattei T, Lehner A, et al. (2006) Deciphering the evolution and metabolism of an anammox bacterium from a community genome. Nature 440: 790-794.

5. Gill SR, Pop M, Deboy RT, Eckburg PB, Turnbaugh PJ, et al. (2006) Metagenomic analysis of the human distal gut microbiome. Science 312: 1355-1359.

6. Turnbaugh PJ, Ley RE, Mahowald MA, Magrini V, Mardis ER, et al. (2006) An obesity-associated gut microbiome with increased capacity for energy harvest. Nature 444: 1027-1031.

7. Garcia Martin H, Ivanova N, Kunin V, Warnecke F, Barry KW, et al. (2006) Metagenomic analysis of two enhanced biological phosphorus removal (EBPR) sludge communities. Nat Biotechnol 24: 1263-1269.

8. Tringe SG, von Mering C, Kobayashi A, Salamov AA, Chen K, et al. (2005) Comparative metagenomics of microbial communities. Science 308: 554-557.

9. Yooseph S, Sutton G, Rusch DB, Halpern AL, Williamson SJ, et al. (2007) The Sorcerer II Global Ocean Sampling Expedition: Expanding the Universe of Protein Families. PLoS Biol 5: e16.

10. Venter JC, Remington K, Heidelberg JF, Halpern AL, Rusch D, et al. (2004) Environmental genome shotgun sequencing of the Sargasso Sea. Science 304: 66-74.
