# Supplementary material for: Global Functional Atlas of Escherichia coli Encompassing Previously Uncharacterized Proteins
Source: PLoS Biol. 2009 Apr 28;7(4):e1000096. doi: 10.1371/journal.pbio.1000096 (PMC2672614; doi:10.1371/journal.pbio.1000096)
Supplement: Protocol S3 — (85 KB DOC) [file pbio.1000096.sd003.doc]

**Protocol S3 – Proteomic analysis**

**Protein complex preparation and characterization by mass spectrometry**

Open reading frames in *E. coli* W3110 were SPA-tagged by targeted homologous recombination using a selectable marker cassette essentially as previously described [1]. Briefly, cell-free soluble protein extracts were prepared by sonication of pelleted cells harvested from log-phase cultures grown in rich (LB) media using standard lysis buffer [1]. Half of each purified protein preparation was subjected to Sodium Dodecyl Sulfate–Polyacrylamide Gel Electrophoresis (SDS-PAGE) followed by silver staining, and the bands were excised from the gel for MALDI-ToF MS peptide mass fingerprinting. The remaining portion was digested in solution with trypsin and analyzed by liquid chromatography-electrospray-linear ion trap tandem mass spectrometry (LCMS).

For the MALDI analysis, after separation by SDS-PAGE and silver staining, the protein bands were excised from the gel with a clean razor. Polypeptides were alkylated and in gel-digested with soluble trypsin. The resulting peptides were extracted with 100 µl of 100 mM NH_4_HCO_3_ and concentrated using bulk C18 reverse phase resin in 384 well collection plates. The peptide mixtures were spotted onto a Bruker MALDI target plate freshly overlayed with 1 µl of R-cyano-4-hydroxycinnamic acid matrix (Fluka Buchs SG, Switzerland). Full scan spectra were acquired on a Reflex IV MALDI-TOF instrument operated in reflectron mode. Automated peak selection and spectral database searches were performed using the Genomics Solutions Knexus package (Discovery Scientific, Inc., Vancouver, Canada). Protein identification was performed using the ProFound search engine, which matches the observed peaks against a database of theoretical peaks, however a Java program developed in-house was used to automate the re-running of Knexus/ProFound using 72 varying parameter sets. The aggregate results were evaluated to calculate a single score for candidate protein identifications. Gel bands were annotated manually using graphical image processing software developed in-house.

For the gel-free shotgun sequencing (LCMS), the protein samples were first dried down from 125 µl to ~20 µl using a Speedvac. An equal volume of digest buffer (50 mM NH_4_ HCO_3_, pH 8.0, 1 mM CaCl_2_) containing a suspension of immobilized trypsin beads (2 µl pre-washed TPCK trypsin, Pierce cat#20230; and 2 µl pre washed Poroszyme trypsin, Applied Biosciences cat# 2-3127-00) was added and the samples incubated with gentle shaking overnight at 30ºC. The peptide mixtures were then acidified with formic acid and loaded using an autosampler onto a 150-μm inner diameter microcapillary fused silica column packed with ~10 cm of reverse phase resin (Zorbax Eclipse XDB-C18; Agilent Technologies, Mississauga, ON) placed in-line with a quaternary HPLC pump and interfaced to an LTQ linear ion trap mass spectrometer (Thermo Finnigan, San Jose, CA). Bound peptides were eluted by electrospray ionization using a 75 min water/acetonitrile gradient with a stable tip flow rate of ~0.250 µl min^-1^. Precursor ions [400-2000 m/z] were subjected to data-dependent, collision-induced dissociation while the mass spectrometer cycled through one full mass scan followed by 11 successive tandem mass scans of the most intense precursor ions with dynamic exclusion enabled. The resulting spectra were matched to candidate peptide sequences by searching against an *E. coli* protein FASTA formatted sequence database using the SEQUEST search algorithm on a cluster computer [SEQUEST-PVM v.27 (rev. 9), with peaklists automatically generated using the embedded ExtractMS script with default parameter settings] [2]. Precursor mass tolerance was set to 3 Da (with daughter mass ion tolerance set to the default of 0), enabling partial tryptic enzyme and single site missed cleavages. The STATQUEST filtering algorithm [3] was then applied to all putative search results to obtain a measure of the statistical reliability (confidence score) for each candidate identification (cutoff *p*-value 0.01, corresponding to a 99% or greater likelihood of being a correct match).

**Gold standards for PI**

Reference datasets were built from experimentally-established physical interactions (positive gold standards) by extracting together pairwise PI interactions obtained from the DIP [4], BIND [5] and INTACT databases [6] after excluding interactions derived from high-throughput experiments previously published by our group [1] and others [7]. To establish a negative gold standard dataset, we compiled protein pairs belonging to different cellular compartments. Specifically, we used putative non-interacting protein pairs wherein existing experimental evidence and or bioinformatic predictions [8] indicates with high confidence that one component is cytoplasmic and the other is associated with the bacterial outer membrane or localized to the periplasm. Inner membrane proteins were discarded in negative gold standard dataset since they are in close physical proximity (and potential physical interaction) with both cytoplasmic and periplasmic proteins.

**Confidence evaluation of PI**

We determined an overall confidence score for each PI generated by the MALDI or LCMS procedures by calculating and integrating the co-purification (CP) scores (defined below) with the primary affinity purification scores using a logistic regression model. Denoting with $X_{ij}^{1}$ and$X_{ij}^{2}$ the CP and primary affinity purification scores respectively, the overall probability of the two proteins interacting,$p_{ij}$, was estimated by:

$$\text{log}it(p_{ij})=\text{ln}(\frac{p_{ij}}{1-p_{ij}})=a+b_{1}X_{ij}^{1}+b_{2}X_{ij}^{2}$$

where $a$, $b_{1},b_{2}$ were learned from the gold standard. The model performance was evaluated through a five-fold cross-validation. We calculated the CP scores within the cross-validation procedure. We also tried building a naïve Bayes integration model, but it was outperformed by the logistic regression model (see **Table S3**). The trained logistic regression models based on the gold standards were then used to assign a single probability value to each of the putative PIs generated in the study.

The CP score provides the relative likelihood of each potential PI by taking bait-prey, bait-bait and prey-prey relationships into account. The CP measure is defined as follows [9]:

Assume $X_{p}=(x_{1p},x_{2p},...x_{Np})$represent the set of protein purifications ($p=1,2,...P$) for *N* proteins, where $x_{ip}$is 1 if the $i^{th}$protein is presented in the$p^{th}$purification and 0 otherwise. We assume that$n_{ij}$,$n_{ii}$and$x_{jj}$are the number of purifications containing both proteins *i* and *j*, either protein *i* or *j*, respectively , so we can define the CP measures as

We also compared the performance of the CP measure defined above with an alternate CP measure [10]:

**Table S3** shows that the Zhang CP measure that we used offered better performance than de Lichtenberg CP measure.

After we processed the PI generated by the MALDI and LCMS procedures, based on the methods described above, we combined them using the method described in **Protocol S6**.

**Determining a suitable cutoff for the high-confidence PI network**

We defined our high confidence physical interaction network based on PIs with predicted probabilities of 0.75 or larger (**Table S6**). Our reasoning for this threshold score is based on the following observations: (i) a high proportion (71%) of PI verified by reciprocal purification (**Table S4**) had likelihood scores at or above this threshold (**Figure S1A**); (ii) the Spearman’s rank correlation coefficient of the 334 reciprocal PIs is 0.774. (iii) at this cutoff the resulting complexes shown a statistically higher functional homogeneity than null random models (**Figure S2**). Finally, the ten most highly connected proteins were further removed for consideration (analogous to the removal of highly promiscuous proteins in a previous large-scale study of yeast protein complexes [11]) based on achieving an optimal functional enrichment of putative protein complexes during clustering (**Protocol S4**).

**References**

1. Butland G, Peregrin-Alvarez JM, Li J, Yang W, Yang X, et al. (2005) Interaction network containing conserved and essential protein complexes in Escherichia coli. Nature 433: 531-537.

2. Eng JK, McCormack AL, Yates JR (2004) An approach to correlate tandem mass spectral data of peptides with amino acid sequences in a protein database. J Am SW Mass Spectrom 5: 976-989.

3. Kislinger T, Rahman K, Radulovic D, Cox B, Rossant J, et al. (2003) PRISM, a generic large scale proteomic investigation strategy for mammals. Mol Cell Proteomics 2: 96-106.

4. Xenarios I, Rice DW, Salwinski L, Baron MK, Marcotte EM, et al. (2000) DIP: the database of interacting proteins. Nucleic Acids Res 28: 289-291.

5. Bader GD, Betel D, Hogue CW (2003) BIND: the Biomolecular Interaction Network Database. Nucleic Acids Res 31: 248-250.

6. Kerrien S, Alam-Faruque Y, Aranda B, Bancarz I, Bridge A, et al. (2007) IntAct--open source resource for molecular interaction data. Nucleic Acids Res 35: D561-565.

7. Arifuzzaman M, Maeda M, Itoh A, Nishikata K, Takita C, et al. (2006) Large-scale identification of protein-protein interaction of Escherichia coli K-12. Genome Res 16: 686-691.

8. Diaz-Mejia JJ, Babu M, Emili A (2009) Computational and experimental approaches to chart the Escherichia coli cell-envelope-associated proteome and interactome. FEMS Microbiol Rev 33: 66-97.

9. Zhang B, Park BH, Karpinets T, Samatova NF (2008) From pull-down data to protein interaction networks and complexes with biological relevance. Bioinformatics 24: 979-986.

10. de Lichtenberg U, Jensen LJ, Brunak S, Bork P (2005) Dynamic complex formation during the yeast cell cycle. Science 307: 724-727.

11. Krogan NJ, Cagney G, Yu H, Zhong G, Guo X, et al. (2006) Global landscape of protein complexes in the yeast Saccharomyces cerevisiae. Nature 440: 637-643.
