# Supplementary material for: Global Functional Atlas of Escherichia coli Encompassing Previously Uncharacterized Proteins
Source: PLoS Biol. 2009 Apr 28;7(4):e1000096. doi: 10.1371/journal.pbio.1000096 (PMC2672614; doi:10.1371/journal.pbio.1000096)
Supplement: Protocol S4 — (34 KB DOC) [file pbio.1000096.sd004.doc]

**Protocol S4** – **Clustering to define complexes, functional modules and neighborhoods**

**Determination of optimal clustering parameters**

Clusters of interacting proteins were generated from three networks: PI (**Protocol S3**), GC (**Protocols S5**) and function prediction (**Protocols S9**) using the Markov Clustering (MCL) algorithm [1], following both biological and structural optimization. These networks are weighted networks, were edge’s weight represent the likelihoods for paired protein interactions in each network. Tuning of cluster granularity in MCL was adjusted by increasing gradually the ‘inflation parameter’, *I*, of MCL. At each value of *I* clusters were drawn, and the optimal *I* (structural optimization) was determined by generating a compromise between the *cluster efficiency* (balance between the probability weights of interaction edges captured within the modules or complexes and the average cluster size) and the *mass fraction* (fraction of interaction edges that connect protein nodes within the same cluster), both of which were tallied using the *clminfo* program [1].

The corresponding clusters were then assessed for functional homogeneity in COGs annotations [2] (biological optimization) using an approach described previously [3]. To determine the significance of functional homogeneity in the resulting clusters, a Z-score was computed using a set of 1000 null models. In these models, the networks remain intact while the COGs annotations were randomly shuffled across proteins. From this procedure, we determined that the optimal MCL inflation parameter for the GC network was 1.7, while the inflation for the PI network was 2.1. In each case the functional homogeneity of the real modules (GC) or complexes (PI) was significantly greater than the null models (Z-score > 15 for the GC network modules, and a Z-score > 3.3 for the PI complexes).

The functional predictions based on computational inference from the integrated (GC+PI) interaction network by machine learning (see **Protocol S9** for details) with existing annotations were clustered to define functional neighborhoods. In this case, we inputted the pairwise Pearson correlation coefficients, computed for every (all-against-all) pairs of vectors of gene function prediction scores and existing annotations (defined as a probability score of 1), to the MCL algorithm, with optimal inflation parameter estimated as described above. A cutoff = 0.5 was used to filter inputted correlation coefficients.

**Graphical visualization of protein complexes and functional modules**

The putative networks of discrete functional modules and protein complexes (shown in **Figures 3E** and **4E**) were visualized using the GenePro plug-in [4] for the Cytoscape software suite [5]. Edges were used to adjoin functional modules and protein complexes with at least 2 connected members in the underlying interaction dataset.

**References**

1. Enright AJ, Van Dongen S, Ouzounis CA (2002) An efficient algorithm for large-scale detection of protein families. Nucleic Acids Res 30: 1575-1584.

2. Tatusov RL, Galperin MY, Natale DA, Koonin EV (2000) The COG database: a tool for genome-scale analysis of protein functions and evolution. Nucleic Acids Res 28: 33-36.

3. Loganantharaj R, Cheepala S, Clifford J (2006) Metric for Measuring the Effectiveness of Clustering of DNA Microarray Expression. BMC Bioinformatics 7 Suppl 2: S5.

4. Vlasblom J, Wu S, Pu S, Superina M, Liu G, et al. (2006) GenePro: a Cytoscape plug-in for advanced visualization and analysis of interaction networks. Bioinformatics 22: 2178-2179.

5. Shannon P, Markiel A, Ozier O, Baliga NS, Wang JT, et al. (2003) Cytoscape: a software environment for integrated models of biomolecular interaction networks. Genome Res 13: 2498-2504.
