# Supplementary material for: Global Functional Atlas of Escherichia coli Encompassing Previously Uncharacterized Proteins
Source: PLoS Biol. 2009 Apr 28;7(4):e1000096. doi: 10.1371/journal.pbio.1000096 (PMC2672614; doi:10.1371/journal.pbio.1000096)
Supplement: Protocol S5 — (72 KB DOC) [file pbio.1000096.sd005.doc]

**Protocol S5 – Prediction of functional interactions by genomic context**

**Generation and evaluation of functional interactions**

**Four** genomic context (GC) methods (Gene Fusions, similarity among Phylogenetic Profiles, conservation of Gene Order, and Intergenic Distances) were used to computationally infer functional associations between pairs of proteins. Homologs and putative orthologs were defined operationally as one-way and bi-directional best-hits (BDBHs) using BLASTP [1], respectively. Briefly, the BLASTP options were default except for a maximum E-value of 1 x 10^-6^, with the database size fixed at 5 x 10^8^, and a final Smith-Waterman alignment covering at least 50% of the shortest sequence. We compared all protein coding genes of *E. coli* K-12 reference sub-strain W3110 against the predicted protein sequences encoded by the other 660 prokaryotic genomes available in the RefSeq database [2] (ftp://ftp.ncbi.nih.gov/genomes/Bacteria/) as of April 2008.

We first built a core set of 440 non-redundant genomes by calculating a Genomic Similarity Score (GSS) based on shared orthologs [3,4], and filtered the full genome dataset to contain genomes with a minimum separation of 0.90 GSS (this threshold filters most of the same-species strains; see [5]). We then proceeded to infer functional interactions.

Gene Fusions were defined by analyses of BLASTP comparisons as reported previously and arbitrarily were considered to have a confidence score of 1.00 [3,4]. Predictions based on Phylogenetic Profiles were obtained by calculating the mutual information of binary vector of presence/absence of homologs or orthologs [5,6]. Mutual information depends on departure of the patterns of conservation of two genes compared to random expectations. We transformed the values of mutual information into a confidence value using the defined gold standards described in the next section. Note, however, that this is not a training step, but rather a transformation of scores prior to integration.

Operon predictions were conducted by two independent method, conservation of Gene Order and Intergenic Distance. Conservation of Gene Order among prokaryotic genomes were performed as reported elsewhere [4]. These predictions do not depend on knowledge of functionally related genes. The confidence values of these interactions depend instead on the assumption that the proportion of conservation of gene order of genes in the same strand compared to the proportion of conserved pairs in opposite strands (representing unrelated genes), represent the biological significance of the conservation. It has been noted that some genes in opposite strands are functionally related, and thus conserved [7,8]. However, the consequence would be an underestimated confidence of our predictions by this method [4,5,9].

Alternativelly, we predicted operons using the Intergenic Distances method [3,10]. This method is the only one out of the four genomic context methods used herein requiring training sets, namely genes reported to belong to the same operons according to literature support in RegulonDB [11], and adjacent genes in the same strand belonging to different transcription units (transcription unit boundaries or TUBs). Despite the lack of any over-fitting effect as previously determined [3,12,13], we further evaluated these predictions using five-fold cross-validation, ensuring that the evaluated pairs were not part of the training sets. That is, the scores for pairs of genes belonging to the gold standards (either negatives or positives) were calculated using training sets lacking such pairs, which were few relative to the total number of predictions (i.e. 729 known pairs in *E. coli* operons, and 555 adjacent pairs in TUBs).

Predicted operons, both by conservation of Gene Order and by Intergenic Distances, across non-redundant prokaryotic genomes, were used to obtain further predictions of functional associations based on operon rearrangements in the Nebulon system [14]. All the four genomic context predictions were integrated to get an overall score using the method discussed in **Protocol S6** , which is the same as the one used to build the STRING database [15]. Despite other genomic context methods use a more liberal cutoff value of 0.7 as a threshold value [15], we opted for a more stringent value of 0.8 to filter out high-confidence associations in our integrated functional association network.

**Gold standards for GC**

To benchmark the functional associations generated by genomic context methods, we built a set of positive and negative gold standards. The positives were defined as pairs of genes belonging to the same pathways reported in EcoCyc [16]. We choose EcoCyc over KEGG [17], because EcoCyc only contains data derived from the literature with supporting experimental characterization in *E.coli*, while KEGG not only contains data derived from homology/orthology propagation, but also it lacks well-defined evidence code tags to differentiate between literature-derived and homology-derived annotations. Negatives were built by combining together assorted pairs of genes whose products participate in different EcoCyc pathways. Pairs of pathways used for this compilation did not have any genes in common (that is, their intersection had to be empty), nor should they generate any pair of genes belonging to the true positive dataset. Please note that to avoid circularity, this set of GC gold standards is independent of both the PI and function prediction gold standards (see **Protocols S3 and S9**, respectively).

**Enrichment analysis of functional interactions**

In order to test the enrichment for functional coherence among the interactions obtained from the genomic context methods, we compared the COGs annotations of interacting partners against 1000 randomly generated null models. In each model networks remained intact, while COG annotations were randomly shuffled across proteins. This procedure ensures that network structure does not influence the statistical assessment of functional coherence.

To assess the statistical significance for enrichment of combination of COGs functional categories (i.e. pairs of proteins with a given pair of COGs), we then calculated a Z-score for each combination of COGs, measured as the number of standard deviations (σ) the observed value (x) is away from the randomly expected mean (μ) from the 1000 null models, following the formula:

Z = (x–μ)/σ

Pairs of COGs showing |Z-score| ≥ 3.3 were considered as statistically significant (p < 0.001), as plotted in **Figure 4D.**

**References**

1. Altschul SF, Madden TL, Schaffer AA, Zhang J, Zhang Z, et al. (1997) Gapped BLAST and PSI-BLAST: a new generation of protein database search programs. Nucleic Acids Res 25: 3389-3402.

2. Pruitt KD, Tatusova T, Maglott DR (2005) NCBI Reference Sequence (RefSeq): a curated non-redundant sequence database of genomes, transcripts and proteins. Nucleic Acids Res 33 Database Issue: D501-504.

3. Moreno-Hagelsieb G, Collado-Vides J (2002) A powerful non-homology method for the prediction of operons in prokaryotes. Bioinformatics 18 Suppl 1: S329-336.

4. Janga SC, Moreno-Hagelsieb G (2004) Conservation of adjacency as evidence of paralogous operons. Nucleic Acids Res 32: 5392-5397.

5. Moreno-Hagelsieb G, Janga SC (2008) Operons and the effect of genome redundancy in deciphering functional relationships using phylogenetic profiles. Proteins 70: 344-352.

6. Huynen M, Snel B, Lathe W, 3rd, Bork P (2000) Predicting protein function by genomic context: quantitative evaluation and qualitative inferences. Genome Res 10: 1204-1210.

7. Korbel JO, Jensen LJ, von Mering C, Bork P (2004) Analysis of genomic context: prediction of functional associations from conserved bidirectionally transcribed gene pairs. Nat Biotechnol 22: 911-917.

8. Overbeek R, Fonstein M, D'Souza M, Pusch GD, Maltsev N (1999) The use of gene clusters to infer functional coupling. Proc Natl Acad Sci U S A 96: 2896-2901.

9. Ermolaeva MD, White O, Salzberg SL (2001) Prediction of operons in microbial genomes. Nucleic Acids Res 29: 1216-1221.

10. Salgado H, Moreno-Hagelsieb G, Smith TF, Collado-Vides J (2000) Operons in Escherichia coli: genomic analyses and predictions. Proc Natl Acad Sci U S A 97: 6652-6657.

11. Salgado H, Gama-Castro S, Peralta-Gil M, Diaz-Peredo E, Sanchez-Solano F, et al. (2006) RegulonDB (version 5.0): Escherichia coli K-12 transcriptional regulatory network, operon organization, and growth conditions. Nucleic Acids Res 34: D394-397.

12. De Hoon MJ, Imoto S, Kobayashi K, Ogasawara N, Miyano S (2004) Predicting the operon structure of Bacillus subtilis using operon length, intergene distance, and gene expression information. Pac Symp Biocomput: 276-287.

13. Price MN, Huang KH, Alm EJ, Arkin AP (2005) A novel method for accurate operon predictions in all sequenced prokaryotes. Nucleic Acids Res 33: 880-892.

14. Janga SC, Collado-Vides J, Moreno-Hagelsieb G (2005) Nebulon: a system for the inference of functional relationships of gene products from the rearrangement of predicted operons. Nucleic Acids Res 33: 2521-2530.

15. von Mering C, Jensen LJ, Snel B, Hooper SD, Krupp M, et al. (2005) STRING: known and predicted protein-protein associations, integrated and transferred across organisms. Nucleic Acids Res 33: D433-437.

16. Keseler IM, Collado-Vides J, Gama-Castro S, Ingraham J, Paley S, et al. (2005) EcoCyc: a comprehensive database resource for Escherichia coli. Nucleic Acids Res 33: D334-337.

17. Kanehisa M, Goto S, Hattori M, Aoki-Kinoshita KF, Itoh M, et al. (2006) From genomics to chemical genomics: new developments in KEGG. Nucleic Acids Res 34: D354-357.
