# Supplementary material for: Global Functional Atlas of Escherichia coli Encompassing Previously Uncharacterized Proteins
Source: PLoS Biol. 2009 Apr 28;7(4):e1000096. doi: 10.1371/journal.pbio.1000096 (PMC2672614; doi:10.1371/journal.pbio.1000096)
Supplement: Protocol S6 — (30 KB DOC) [file pbio.1000096.sd006.doc]

**Protocol S6 – Global integration of different data sources for function prediction**

In practice, we assume the reliabilities of the associations generated by different experimental and computational sources are independent. Therefore, we computed the integrated weight score $a{}_{ij}$ for a functional association between protein *i* and protein *j* as follows [1]:

$a{}_{ij}=1-\prod_{v=1}^{k} (1-a_{ij}^{v})$,

where $a_{ij}^{v}$ ($0\leq a_{ij}\leq1$) is the estimated weight score of the interaction between *i* and *j* in data source *v*, and *k* is the number of data sources the interaction was found. The method treats each $a{}_{ij}$ as a probability. Similar methods have been used by other groups to integrate different functional association evidence [2,3]. For this study, the formula was used to integrate the following networks: (1) a high confidence PI network by combining the interactions generated by LCMS and MALDI methods (**Protocol S3**); (2) a unified GC network combining interactions from the four genomic context methods (**Protocol S5**); and (3) a fully integrated interaction network by integrating the PI and GC networks for network-based function prediction (**Protocol S9**).

**References**

1. von Mering C, Jensen LJ, Snel B, Hooper SD, Krupp M, et al. (2005) STRING: known and predicted protein-protein associations, integrated and transferred across organisms. Nucleic Acids Res 33: D433-437.

2. Chua HN, Sung WK, Wong L (2006) Exploiting indirect neighbours and topological weight to predict protein function from protein-protein interactions. Bioinformatics 22: 1623-1630.

3. Nabieva E, Jim K, Agarwal A, Chazelle B, Singh M (2005) Whole-proteome prediction of protein function via graph-theoretic analysis of interaction maps. Bioinformatics 21 Suppl 1: i302-310.
