# Supplementary material for: Global Functional Atlas of Escherichia coli Encompassing Previously Uncharacterized Proteins
Source: PLoS Biol. 2009 Apr 28;7(4):e1000096. doi: 10.1371/journal.pbio.1000096 (PMC2672614; doi:10.1371/journal.pbio.1000096)
Supplement: Protocol S7 — (28 KB DOC) [file pbio.1000096.sd007.doc]

**Protocol S7 – Analysis of topological network properties**

We generated a fully integrated interaction network by combining the high confidence PI (**Protocol S3**) and GC network (**Protocol S5**) using the scoring approach described in **Protocol S6**. To examine the topological properties of this integrated network and the PI and GC networks, we used *igraph*, a publicly available R package for creating, manipulating and analyzing graphs (<http://cneurocvs.rmki.kfki.hu/igraph/>). In particular, since all the graphs analyzed in this study are undirected, we used the corresponding versions of the functions: *degree, transitivity* and *shortest paths,* for calculating the degree, clustering coefficient, shortest path between any pair of nodes, respectively. Additionaly, *betweenness*, a measure of centrality of a node, denoting the number of shortest paths going through a node, was calculated using the *brandes* algorithm [1] implemented in *igraph*. Similarly, closeness, which reflects the average length of the shortest paths to all the other vertices in the graph, was obtained using *igraph*. Since the centrality measures, betweenness and closeness, use the shortest path lengths between all pairs of nodes in a graph, for cases where no path exists between a particular pair of nodes, the shortest path length was taken as 1 minus the maximum number of nodes in the network. Note that this is also the default assumption for calculating centrality measures in *igraph*.

To assess the significance of the observed network property for orphans compared to annotated genes, we compared their values against a collection 1,000 sets of randomly selected annotated genes from the respective network each containing the same number of proteins than the orphan’s set, and estimated *p*-values based on this comparison.

**References**

1. Brandes U (2001) A Faster Algorithm for Betweenness Centrality. Journal of Mathematical Sociology 25: 163-177.
