# Supplementary material for: Global Functional Atlas of Escherichia coli Encompassing Previously Uncharacterized Proteins
Source: PLoS Biol. 2009 Apr 28;7(4):e1000096. doi: 10.1371/journal.pbio.1000096 (PMC2672614; doi:10.1371/journal.pbio.1000096)
Supplement: Protocol S8 — (34 KB DOC) [file pbio.1000096.sd008.doc]

**Protocol S8 – Calculating node similarity in the integrated functional association network**

Previous studies have shown that the topological structure of a node (gene product) in a functional association network is informative for protein function prediction [1] and functional module inference [2,3]. We therefore applied an established topological overlap measure (TOM score), originally proposed by Ravasz et al. [2] for binary networks and later extended by Zhang et al. [3] to define the similarity of pairs of nodes in weighted networks:

$w_{ij}=\frac{l_{ij}+a_{ij}}{\text{min}\left\{m_{i},m_{j}\right\}+1-a_{ij}}$ , $0\leq w_{ij}\leq1$

where $0\leq a_{ij}\leq1$ is the weight of a function association between protein *i* and *j*, $l_{ij}=\sum_{u\neq i,j} a_{iu}a{}_{uj}$, $m_{i}=\sum_{u\neq i} a_{iu}$and $m_{j}=\sum_{u\neq j} a_{uj}$is the connectivity of nodes *i* and *j.* This quantitative measure was used to define the relative interconnectedness of two gene products in our integrated functional association network.

**References**

1. Chua HN, Sung WK, Wong L (2006) Exploiting indirect neighbours and topological weight to predict protein function from protein-protein interactions. Bioinformatics 22: 1623-1630.

2. Ravasz E, Somera AL, Mongru DA, Oltvai ZN, Barabasi AL (2002) Hierarchical organization of modularity in metabolic networks. Science 297: 1551-1555.

3. Zhang B, Horvath S (2005) A general framework for weighted gene co-expression network analysis. Stat Appl Genet Mol Biol 4: Article17.
