# Supplementary material for: Global Functional Atlas of Escherichia coli Encompassing Previously Uncharacterized Proteins
Source: PLoS Biol. 2009 Apr 28;7(4):e1000096. doi: 10.1371/journal.pbio.1000096 (PMC2672614; doi:10.1371/journal.pbio.1000096)
Supplement: Protocol S9 — (154 KB DOC) [file pbio.1000096.sd009.doc]

**Protocol S9 – Network-based protein function prediction**

**Gold standards for function predictions**

We created three gold standards for the function predictions based on Gene Ontology (GO) [1,2], COGs functional annotations [3] and MultiFun databases; [4] (**Table S14**). We used these gold standards in order to have a greater coverage of protein function, rather than using a single annotation source. We expect that these databases have a considerable overlap by historical reasons; but also we consider that their respective curation particularities are useful in function prediction. In order to have a suitable number of proteins for cross-validation, we only considered those functional categories with the minimum of 15 associated labeled proteins. Since some functional term are too general if (i.e. thay have too many labeled proteins), we also fixed the maximum number of labeled proteins in a function term to be 400. Suitable gold standards were carefully selected to benchmark our predictions. Specifically, for each of the three gold standards, we selected the functional terms as follows:

**GO**: We focused on 51 biological process (BP) terms from the GO hierarchy (<http://www.ebi.ac.uk/GOA/>), removing GO terms used for building classification models that had either IPI (inferred from protein-protein interaction) nor IGC (inferred from genomic context) evidence codes. We removed any proteins with NCBI product descriptions (**Table S14)** as “hypothetical”, “predicted” or “putative”.

**MultiFun**: We selected 19 middle-level MultiFun functional annotation terms available through the MultiFun (GeneProtEC) public database. We removed any proteins with NCBI product descriptions (**Table S14**) as “hypothetical”, “predicted” or “putative”, since any functional annotations for these proteins are potentially unreliable.

**COGs**: We discarded COGs functional categories “R” and “S” since they refer to uninformative “General function prediction only” and “Function unknown” annotations, respectively. For the remaining 18 terms, we also removed proteins with an NCBI product (**Table S14**) description as “hypothetical”, “predicted” or “putative”. It is worth noting, that the seminal COGs classifications were constructed using BLAST to look for groups of ortholog sequences, constructed with a series of homology-based methods and verifications of ortholog consistency and were manually curated to assign each COG to discrete functional categorical terms, similar to MultiFun terms devised by Riley and colleagues. But later, COGs are inferred by homology/orthology alone from preexisting groups.

**Algorithm**

Supervised computational methods for automatically assigning functional terms to previously uncharacterized genes based on the categorical properties of their annotated interaction partners have been developed [5-7]. These can be roughly categorized as either simple, local, guilt-by-association techniques or global, network optimization procedures. While both approaches often achieve similar performance [8], there are potential deficiencies in procedures that ignore the overall network structure since the topological properties can be functionally highly informative [9-11]. Moreover, since functional terms are often interrelated (e.g. the GO and MultiFun hierarchies), the correlation structure of the respective functional categories can potentially be exploited. Hence, to deduce the biological role(s) of a particular protein, one should take into account the full spectrum and relatedness of available annotations of the interaction partners when evaluating a particular functional category.

For this study, we developed a new network-based protein function prediction method that assign functions to unannotated orphan proteins using the functional information from their first-order (direct) neighbors and second-order (indirect) neighbors in our integrated functional association network (see the example discussed below). The algorithm is summarized as follows:

Let us assume to have *N,* (*i=1, 2,…,N*), proteins in the integrated network, of which $N_{l}$ ($N_{l}<N$) have labels representing *M* distinct functional categories while the other *N-*$N_{l}$are unannotated. Our function prediction algorithm consists of two steps:

1. For each protein *i*, and each functional category H, we compute the affinity vector of the protein for that function. Such vector is constituted by two elements, containing the scores of function *H* for protein *i* based on information from its first- and second-order neighbors (see details below).
2. For each protein i, we then compute its probability of having function *H* based on the penalized logistic regression model [12].

In the following section, we discuss the details of each step:

**Step 1) Computation of Affinity Vectors**

Let us assume$N_{i}^{*}$ to be the set of first-order neighbors of protein *i*. The neighbors include at least one of three types of proteins: proteins labeled (annotated) as function *H*, proteins labeled as other functions excluding *H,* and proteins without any functional annotations (uncharacterized orphans) (see the example discussed **below**). Let us call$N_{i}$($N_{i}\in N_{j}^{*},j\neq i$) the subset of first-order neighbors of protein *i* which have a functional label (annotated). Moreover, we introduce a discrete value function$\theta(j,H)$, where $j\in N_{i}^{*}$, which takes value 1 if protein *j* has function *H*, and 0 otherwise. The first-order function score $f^{1st}(i,H)$of protein *i* for function *H* is given by:

$$f^{1st}(i,H)=\frac{\sum_{j\in N_{i}} w_{ij}*\theta(j,H)}{\sum_{j\in N_{i}} w_{ij}}$$

Let $B_{1},B_{2},...,B_{n}$be the first-order neighbors of protein *i* and let us indicate their first-order scores for function H with $f^{1st}(B_{1},H)$,$f^{1st}(B_{2},H)$,…,$f^{1st}(B_{n},H)$. The second-order function score $f^{2st}(i,H)$ of protein *i* for function *H* is then given by:

$$f^{2st}(i,H)=\frac{\sum_{j\in N_{i}^{*}} f^{1st}(B_{j},H)*\theta(j,H)}{\sum_{j\in N_{i}^{*}} f^{1st}(B_{j},H)}$$

The affinity vector of protein *i* for function *H* is then constituted by:

$$f(i,H)=[f^{1st}(i,H),f^{2st}(i,H)]$$

An example of the process for constructing the affinity vector of a given protein *v* assigned to function *H* is given in the below Example section:

**Step 2) Probabilistic Functional assignment**

We model the probability of assigning proteins *i* to function *H* based on the penalized logistic regression model [12]:

To estimate the logistic regression coefficients, we minimized the following equation:

where $l$indicates the binomial log-likelihood and $\lambda$is a positive constant.

When the number of functions considered in a study is large, the model may include many parameters. For example, we used 51 GO functions in this study, so the number parameters are 51*2+1=103. However, it is also well-known that some functions are correlated. Therefore, a feature selection procedure is necessary to reduce the number of parameter estimations in the modeling procedure. We used a stepwise variable selection procedure [12], that is, first run a forward selection, then do a backward deletion. In each forward step, a feature is added to the model. A preset number of forward steps are repeated. In the subsequent backward steps, a redundant feature is deleted, beginning with the final model from the forward steps. The procedure-based penalized logistic regression models are implemented in stepPlr R package [12]. Therefore, we named our method as **StepPLR**.

**Example**

Here we provide an example illustrating the construction of the affinity vector of a given protein *v* assigned to function *H*:

0.9

Different colours are used to represent different protein functions: Black indicates unannotated proteins. Ideally, multiple colours should be provided for proteins with multiple functions. However, to simplify this visualization, we show only one colour for each protein. In reality, we computed different affinity vectors if a given protein is annotated with more than one functions. The assumed value assigned to each edge is the calculated pairwise node integrated network topological overlap measure (TOM), described in **Protocol S8.**

Therefore, we can construct the affinity vector $f(v,red)$of the orphan protein *v* with function *H* (red) as:

1. Compute the function score $f^{1st}(v,red)$using *v*’s first order neighbours. We only focus on the annotated proteins **B1**, **B2, B3** and **B4**, which have TOM values 0.4, 0.9, 0.8 and 0.1, respectively. Proteins B2 and B3 are annotated with red function while proteins B1 and B4 are annotated as other functions. We have

$f^{1st}(v,red)$=(0.9+0.8)/(0.4+0.9+0.8+0.1)=0.77

1. Compute the function score $f^{2st}(v,red)$using v’s second order neighbours. Protein *v* has five proteins B1, B2, B3, B4 and B5 in its first-order neighbours. We compute their respective function scores $f^{1st}(B1,red),$ $f^{1st}(B2,red),$ $f^{1st}(B3,red),$ $f^{1st}(B4,red)$and $f^{1st}(B5,red)$ in their first-order neighbours as

$f^{1st}(B1,red)$=1/1=1; $f^{1st}(B2,red)$=(0.1+0.8)/(0.2+0.1+0.8)=0.82

$f^{1st}(B3,red)$=0/0.5=0; $f^{1st}(B4,red)$=0/0.6=0

$f^{1st}(B5,red)$=0/0.5=0

Therefore we have

$f^{2st}(v,red)=(f^{1st}(B2,red)+f^{1st}(B3,red))/(f^{1st}(B1,red),f^{1st}(B2,red),f^{1st}(B3,red),f^{1st}(B4,red),f^{1st}(B5,red))$ =(0.82+0)/(1+0.82+0+0+0)=0.45

Therefore, we construct the affinity vector of protein *v* with function *H* (red) as

$$f(v,H)=f(v,red)=\{f^{1st}(v,red),f^{2st}(v,red)\}=\{0.77,0.45\}$$

**Evaluation of function predictions**

Classifier performance of each functional term was evaluated by five-fold cross-validation and quantified based on the area-under-the-curve (AUC) obtained for each classifier term in Receiver Operating Characteristic (ROC) curves drawn by plotting sensitivity versus specificity or precision to evaluate the performance of our computational method in the cross-validation procedure for each functional class of the three functional classification schemas (COGs, GO, MultiFun).

To evaluate the overall prediction performance of our algorithm, we plotted the precision against recall at different threshold confidence cutoffs for each functional classification schemas, which can be calculated as follows: Given a threshold *c*, if a protein has predicted probability larger than c in a function category, we assigned the function to the protein, so we compute

$precision=\sum_{i==1}^{n} k_{i}/\sum_{i==1}^{n} m_{i}$ , $recall=\sum_{i==1}^{n} k_{i}/\sum_{i==1}^{n} n_{i}$

where $k_{i}$ is the number of functions correctly predicted for protein *i*; $m_{i}$ is the number of functions predicted for protein i, $n_{i}$ is the number of functions annotated for protein *i*. Each threshold yields one pair of sensitivity and specificity values and, thus, one point on the curve. Such analyses can be summarized with a single statistic – area-under-the-curve (AUC) – which provides a quantitative indication of how well a particular functional classifier performs. The results are plotted in **Figure S4B** and summarized in a tabular manner in **Table S15.**

In general, we observed that classifiers for metabolism-related functions offered better performance than the others. In particular, the performances of classifiers for similar function terms in GO, COGs and MultiFun exhibited consistent trends. Conversely, many of the functions with relatively lower AUC values included proteins participating in different pathways. For example, the classifiers for cell wall in COGs and GO have relatively high AUC value (~0.84) while those for transcription have relatively worse performance (AUC: 0.73-0.75).]

**References**

1. Ashburner M, Ball CA, Blake JA, Botstein D, Butler H, et al. (2000) Gene ontology: tool for the unification of biology. The Gene Ontology Consortium. Nat Genet 25: 25-29.

2. Camon E, Magrane M, Barrell D, Lee V, Dimmer E, et al. (2004) The Gene Ontology Annotation (GOA) Database: sharing knowledge in Uniprot with Gene Ontology. Nucleic Acids Res 32: D262-266.

3. Tatusov RL, Koonin EV, Lipman DJ (1997) A genomic perspective on protein families. Science 278: 631-637.

4. Serres MH, Goswami S, Riley M (2004) GenProtEC: an updated and improved analysis of functions of Escherichia coli K-12 proteins. Nucleic Acids Res 32: D300-302.

5. Nabieva E, Jim K, Agarwal A, Chazelle B, Singh M (2005) Whole-proteome prediction of protein function via graph-theoretic analysis of interaction maps. Bioinformatics 21 Suppl 1: i302-310.

6. Schwikowski B, Uetz P, Fields S (2000) A network of protein-protein interactions in yeast. Nat Biotechnol 18: 1257-1261.

7. Vazquez A, Flammini A, Maritan A, Vespignani A (2003) Modeling of protein interaction networks. Complexus 1: 38-44.

8. Murali TM, Wu CJ, Kasif S (2006) The art of gene function prediction. Nat Biotechnol 24: 1474-1475; author reply 1475-1476.

9. Chua HN, Sung WK, Wong L (2006) Exploiting indirect neighbours and topological weight to predict protein function from protein-protein interactions. Bioinformatics 22: 1623-1630.

10. Ravasz E, Somera AL, Mongru DA, Oltvai ZN, Barabasi AL (2002) Hierarchical organization of modularity in metabolic networks. Science 297: 1551-1555.

11. Zhang B, Horvath S (2005) A general framework for weighted gene co-expression network analysis. Stat Appl Genet Mol Biol 4: Article17.

12. Park MY, Hastie T (2007) Penalized logistic regression for detecting gene interactions. Biostatistics 9: 30-50.
