# Supplementary material for: Global Functional Atlas of Escherichia coli Encompassing Previously Uncharacterized Proteins
Source: PLoS Biol. 2009 Apr 28;7(4):e1000096. doi: 10.1371/journal.pbio.1000096 (PMC2672614; doi:10.1371/journal.pbio.1000096)
Supplement: Protocol S10 — (33 KB DOC) [file pbio.1000096.sd010.doc]

**Protocol S10** – **Comparison of our algorithm (StepPLR) with established methods**

Our function prediction method is a network neighborhood-based method, in that the function of an unannotated orphan is determined based on the available annotations of its direct and indirect neighbors. We compared the prediction performance of our method (StepPLR) with other representative methods: Majority counting, Weighted majority, Chi-squared-1st and Chi-squared-2nd (1^st^ and 2^nd^ neighbor, respectively):

**Majority counting method**: As proposed by Schwikowski and colleagues [1], we considered all labeled proteins in the first-neighbors of a given protein *v* and count the number of times ($n_{v}(H)$) function *H* occurs for the protein. For a weighted functional association network, the function score of a given protein *v* with function *H* can be computed based on formula to calculate $f^{1st}(i,H)$ in **Protocol S9**. A similar idea was developed by McDemott et al. [2], although they used a slightly different way to define denominator of the formula for calculating $f^{1st}(i,H)$ as a source factor for adjusting the contribution of predicted interactions derived from public databases. We named the majority counting method as “Majority” and the weighted method as “Weighted Majority” methods.

**Chi-squared method**: We evaluated the method proposed by Hishigaki et al. [3]. The approach compute a chi-square statistic for function *H* in the neighbors of a given protein *v* as

where $n_{v}(H)$is the number of times function *H* occurs for protein *v* in the *vth* order neighbor and $e_{v}(H)$is the expected number of times function *H* occurs for protein *v* in the *vth* order neighbor. We considered both the first and second order neighbors, naming these two procedures as Chi-Squared1st and Chi-Squared2nd, respectively.

**Performance evaluation**

We used a training and test strategy; that is, we sampled and reserved a portion of labeled data as a test set and used the other labeled data as training data. We then built the prediction models on the training data and used the test data to evaluate the performance of the prediction methods based on precision and recall (see **Protocol S9**) calculated at different probability level. The results are shown in **Figure S4A**. In all these cases, our new method generated better prediction performance than the other four computational methods. Similar results were also obtained for the COGs and GO functional classification categories (data not shown).

**References**

1. Schwikowski B, Uetz P, Fields S (2000) A network of protein-protein interactions in yeast. Nat Biotechnol 18: 1257-1261.

2. McDermott J, Bumgarner R, Samudrala R (2005) Functional annotation from predicted protein interaction networks. Bioinformatics 21: 3217-3226.

3. Hishigaki H, Nakai K, Ono T, Tanigami A, Takagi T (2001) Assessment of prediction accuracy of protein function from protein--protein interaction data. Yeast 18: 523-531.
