# Supplementary material for: Global Functional Atlas of Escherichia coli Encompassing Previously Uncharacterized Proteins
Source: PLoS Biol. 2009 Apr 28;7(4):e1000096. doi: 10.1371/journal.pbio.1000096 (PMC2672614; doi:10.1371/journal.pbio.1000096)
Supplement: Protocol S11 — (47 KB DOC) [file pbio.1000096.sd011.doc]

**Protocol S11 – Experimental validations of functional predictions**

**Drug screening**

*E. coli* non-essential single gene deletion mutant strains, obtained from the “Keio” collection [1], and potentially hypomorphic SPA-tagged essential *E. coli* proteins [2] were evaluated for sensitivity to various antibiotics that inhibit cell wall and protein translation. The strains were pinned onto the rich Luria Bertani (LB) media plates in the absence or presence of drugs inhibiting cell wall assembly or protein translation. Reproducibility was ensured by pinning each strain four times per plate. After 24 h of incubation at 32 °C, the colonies from each plate were digitally imaged using a high resolution digital camera (Canon Powershot A640, 10 Megapixels). Colony sizes were analyzed statistically to take into account systematic errors (e.g. plate edge effects), data reproducibility and deviations from the mean colony sizes using an in house automated image processing system [3].

For each antibiotic (*A*), the colony sizes of each mutant (*i*) were determined both with and without *A* and compared using the formula Log2(Q/R), where Q is the size of the resulting colony in LB medium supplemented with *A*, and R is the size of the mutant in LB medium without *A*. Obtained Log2 ratios were normalized using the average (μ) and standard deviation (σ) of a genomic pool of strains (Keio collection) exposed or not to *A* to obtain the corresponding Z-score:

The resulting Z-scores for relevant orphans and a few randomly selected controls are shown in **Figures 5C and 6E**.

**Functional enrichment analysis of drug screen results**

The extracted raw colony sizes from each genome-wide drug screen were subjected to gene set analysis (GSA) [4], implemented in the R package (http://www-stat.stanford.edu/~tibs/GSA/), to determine statistical functional enrichment among the annotated and orphan gene sets putatively linked to either cell wall assembly or protein translation (see **Table S18**). The annotated gene sets were represented by the positive gold standards generated for the COGs function terms “M -Cell wall/membrane/envelope biogenesis” and “J-Translation/ribosomal structure/and biogenesis”. The orphan represented unannotated proteins with functional prediction probability scores larger than 0.8 or with a maximum probability score assigned to either of these two functions among all possible COG terms (**Table S16**). The results are shown in **Table S18**).

**Translational defect assays**

β-gal translation fidelity assays were performed in octuplicate essentially as described [5] using previously reported expression plasmids [6], with the exception that the *E. coli* cells were grown at 30 ºC and the β-lactamase activities were measured using a CENTA kit (Calbiochem). Global translation efficiency was determined in quadruplicate by measuring the incorporation of ^35^S methionine into total proteins synthesized for 1 hr in exponentially growing cells. Ribosome profiles were analysed essentially as described [7]. In brief, cell extracts were prepared from fresh cultures grown to OD_600_ 0.5-0.8 at 30 ^o^C. Pre-cleared cell extracts were applied to a sucrose gradient 10-40% (w/v) and ultracentrifuged at 35,000 rpm for 3 h in a Beckman SW40Ti rotor. Aliquots (0.1 ml) of the resulting gradients were then collected from bottom to top and analysed spectrophotometrically at 254 nm. Each experiment was repeated three times with similar results.

**Crystal violet-based assay for testing biofilm formation** Ability of the *E. coli* gene deletion mutant strains (Keio collection) defective for biofilm formation was assayed by crystal violet staining, essentially as described previously [8]. The overnight cultures grown in LB or CFA medium at 32 °C were diluted by 1:10 to make a volume of 100 μl in a 96-well polystyrene microtiter plate (Corning Inc., NY, USA). After incubating the plates for periods of 24 and 48 h, the medium was discarded and individual wells were stained with 130 μl of 0.1% crystal violet (CV, Sigma) solution. The plate was incubated at room temperature for 30 min. The CV solution was removed and washed forcefully with deionized water until all the unbound CV solution was successfully removed. Plates were then incubated at 32 °C for 20 min after which the wells were filled with 150 μl of 95% ethanol and incubated for 10 minutes at room temperature. The amount of cells attached to the surface, reflecting the formation of biofilm was measured by reading the absorbance at an optical density of 600nm. Each strain was grown in four different wells per experiment. Average intensity of the biofilm formation for each deletion mutant was compared to the wild type control. The average intensity of the biofilm formation for each deletion mutant is generated from eight independent replicate experiments.

**Auxotrophy growth assays**

Auxanography was performed as reported previously [9] by incorporating 10^7^ deletion mutant cells into a 3-ml of 0.6% soft agar layer on glucose (15 g/l) limited minimal media agar plates. Sterile 1.5 mm filter paper discs were dipped into varying amounts of shikimic acid or aromatic amino acid mixtures (1mM phenylalanine and tyrosine, 0.5mM tryptophan, 0.1mM *p-*aminobenzoic acid, 0.4mM *p*-hydroxybenzoic acid). The paper discs were then placed onto the surface of the soft agar medium, and the plates were incubated for 72 h at 32 °C for the appearance of zones of growth. The zone of growth was not proportional to the varying concentrations of shikimic or aromatic amino acid tested.

**References**

1. Baba T, Ara T, Hasegawa M, Takai Y, Okumura Y, et al. (2006) Construction of Escherichia coli K-12 in-frame, single-gene knockout mutants: the Keio collection. Mol Syst Biol 2: 2006 0008.

2. Butland G, Peregrin-Alvarez JM, Li J, Yang W, Yang X, et al. (2005) Interaction network containing conserved and essential protein complexes in Escherichia coli. Nature 433: 531-537.

3. Butland G, Babu M, Diaz-Mejia JJ, Bohdana F, Phanse S, et al. (2008) eSGA: E. coli synthetic genetic array analysis. Nat Methods 5: 789-795.

4. Efron B, Tibshirani R (2007) On testing the significance of sets of genes. Ann Appl Stat 1: 107-129.

5. O'Connor M, Goringer HU, Dahlberg AE (1992) A ribosomal ambiguity mutation in the 530 loop of E. coli 16S rRNA. Nucleic Acids Res 20: 4221-4227.

6. Thompson J, O'Connor M, Mills JA, Dahlberg AE (2002) The protein synthesis inhibitors, oxazolidinones and chloramphenicol, cause extensive translational inaccuracy in vivo. J Mol Biol 322: 273-279.

7. Charollais J, Pflieger D, Vinh J, Dreyfus M, Iost I (2003) The DEAD-box RNA helicase SrmB is involved in the assembly of 50S ribosomal subunits in Escherichia coli. Mol Microbiol 48: 1253-1265.

8. Pratt LA, Kolter R (1998) Genetic analysis of Escherichia coli biofilm formation: roles of flagella, motility, chemotaxis and type I pili. Mol Microbiol 30: 285-293.

9. Lobner-Olesen A, Marinus MG (1992) Identification of the gene (aroK) encoding shikimic acid kinase I of Escherichia coli. J Bacteriol 174: 525-529.
