# Supplementary material for: Global Functional Atlas of Escherichia coli Encompassing Previously Uncharacterized Proteins
Source: PLoS Biol. 2009 Apr 28;7(4):e1000096. doi: 10.1371/journal.pbio.1000096 (PMC2672614; doi:10.1371/journal.pbio.1000096)
Supplement: Protocol S12 — (22 KB DOC) [file pbio.1000096.sd012.doc]

**Protocol S12 – Analysis of functional interactions of orphans extend beyond Proteobacteria**

Putative orthologs of orphans and annotated genes were determined for the protein components of specified functional neighborhoods by BLAST BDBHs (see **Protocol S5**). The proportion of genomes containing putative orthologs in each phylogenetic taxon was computed for each neighborhood. Values shown in **Figure 7A** correspond to the number of genomes with orthologs of pairs of proteins (either annotated-annotated, annotated-orphan or orphan-orphan) for each taxon. **Figure 7B** corresponds to the number of genomes with orthologs of proteins of either orphans or annotated genes for each taxon, divided by the total number of genomes in such taxon. This procedure pre-filtered redundant strains (those showing a high amount of highly similar sequences) as described before [1].

**References**

1. Moreno-Hagelsieb G, Janga SC (2008) Operons and the effect of genome redundancy in deciphering functional relationships using phylogenetic profiles. Proteins 70: 344-352.
