# Supplementary material for: Global Functional Atlas of Escherichia coli Encompassing Previously Uncharacterized Proteins
Source: PLoS Biol. 2009 Apr 28;7(4):e1000096. doi: 10.1371/journal.pbio.1000096 (PMC2672614; doi:10.1371/journal.pbio.1000096)
Supplement: Protocol S13 — (38 KB DOC) [file pbio.1000096.sd013.doc]

**Protocol S13 – Public web server details**

A dedicated web server ‘eNet’ (http://ecoli.med.utoronto.ca) for the entire collection of high confidence *E. coli* physical interactions, genomic context-based protein functional interaction predictions, and orphan function predictions, has been implemented and is freely accessible. All the supplementary tables and figures pertaining to this manuscript can be downloaded via this website (“Downloads” section). The site has been designed with an intuitive user interface which allows searches based on single or multiple proteins (by gene name or accession/b-numbers), keywords or via batch text file download. Examples of search modes are provided in the “Help” section of this website. Users can browse the supporting PI and GC binary interactions, along with the function predictions generated for the orphans in this study together with the existing gene annotations of previously characterized genes. The website also provides links to many of the major public databases, including sources used in this study. A detailed view of the prediction profile for a gene with new functions and predictions that match the current annotations highlighted is also available.
