# Supplementary material for: Global Functional Atlas of Escherichia coli Encompassing Previously Uncharacterized Proteins
Source: PLoS Biol. 2009 Apr 28;7(4):e1000096. doi: 10.1371/journal.pbio.1000096 (PMC2672614; doi:10.1371/journal.pbio.1000096)
Supplement: Table S3 — (8 KB PDF) [file pbio.1000096.st003.pdf]

Table S3. Performance comparison of different methods in PI analysis.

	Accuracies of classification model [a]	
Co-purification measure [b]	Logistic regression model	Naive Bayes model
Zhang measure [c]	0.85[e] (0.82[f])	0.82 (0.81)
de Lichtenberg measure [d]	0.82 (0.80)	0.79 (0.76)

[a] The accuracies were calculated based on five-fold cross-validation procedure.
[b] This is the covariate X_{ij}^{1} used in the logistic regression model of Section **Confidence evaluation of PI** in Protocol S3.
[c] The is the method developed by Zhang et al. (2008) in Section **Confidence evaluation of PI** in Protocol S3.
[d] The is the method developed by de Lichtenberg et al. (2005)) in Section **Confidence evaluation of PI** in Protocol S3.
[e] Accuracy based on LCMS data.
[f] Accuracy based on MALDI data.
