# Supplementary material for: Global Functional Atlas of Escherichia coli Encompassing Previously Uncharacterized Proteins
Source: PLoS Biol. 2009 Apr 28;7(4):e1000096. doi: 10.1371/journal.pbio.1000096 (PMC2672614; doi:10.1371/journal.pbio.1000096)
Supplement: Table S5 — (7 KB PDF) [file pbio.1000096.st005.pdf]

Table S5. Promiscuous ‘hub’ proteins filtered from the PI dataset

Interactions	Gene name	Bnumber	JW number	Function
225	aceE	b0114	JW0110	pyruvate dehydrogenase, decarboxylase component E1, thiamin-binding
201	aceF	b0115	JW0111	pyruvate dehydrogenase, dihydrolipoyltransacetylase component E2
178	dnaK	b0014	JW0013	chaperone Hsp70, co-chaperone with DnaJ
170	tufA	b3339	JW3301	protein chain elongation factor EF-Tu
155	rpsE	b3303	JW3265	30S ribosomal subunit protein S5
154	lpd	b0116	JW0112	lipoamide dehydrogenase, E3 component is part of three enzyme complexes
152	rplV	b3315	JW3277	50S ribosomal subunit protein L22
148	rplC	b3320	JW3282	50S ribosomal subunit protein L3
122	rpsB	b0169	JW0164	30S ribosomal subunit protein S2
115	rpsG	b3341	JW3303	30S ribosomal subunit protein S[illegible]
