# Supplementary material for: Global Functional Atlas of Escherichia coli Encompassing Previously Uncharacterized Proteins
Source: PLoS Biol. 2009 Apr 28;7(4):e1000096. doi: 10.1371/journal.pbio.1000096 (PMC2672614; doi:10.1371/journal.pbio.1000096)
Supplement: Table S11 — (8 KB PDF) [file pbio.1000096.st011.pdf]

Table S11. Comparison of PI Vs GC interactions and modules

	GC interactions [a]				GC modules [b]			
	GC all (this study) [c]	GC afp (this study) [d]	STRING all [e]	STRING gc [f]	GC all (this study)	GC afp (this study)	STRING all	STRING gc
PI (this study)	399(6.6%)	149(2.4%)	593(9.8%)	201(3.35%)	16.91	31.35	43.28	44.31
PI low-thoroughput [g]	610(39.1%)	368(23.6%)	1202(77.1%)	502(32.2%)	63.82	119.62	174.28	163.54
Arifuzzaman	437(3.9%)	121(1.0%)	314(2.8%)	229(2.05%)	6.90	12.76	10.59	12.21
Rain (*H. pylori*) [h]	46(3.3%)	ND	39(2.8%)	ND	11.35	ND	11.74	ND

[a] Overlapping edges between PI and GC networks. Values indicate the number of interactions overlapping between any pair PI/GC networks and the percentage of interactions that this overlap represents with respect to the total number of PI (in parenthesis)

[b] Mapping of PI edges onto GC modules. The real frequencies of PI whose two interactors belong to the same GC module were compared against 1000 null models to obtain a Z-score = (Nreal-Nrand)/Stdev_rand. Null models represent randomly rewired PI networks as described before (Maslov and Snepeen 2002). Values represent obtained Z-scores.

[c] GC all: All genomic context methods used in this study

[d] GC afp: All genomic context methods used in this study but excluding “operon rearrangements” (to make a set equivalent to GC methods used in STRING database)

[e] STRING all: All interactions reported for *E. coli* W3110 in STRING database (these includes non-GC methods like text-minning, and experimental data, like PI and co-expression)

[f] STRING gc: All GC methods reported in STRING database (analog to GC afp)

[g] *E. coli* PI from DIP, BIND and IntAct excluding data from Butland et al. (2005) and Arifuzzaman et al. (2006)

[g] *H. pylori* PI from Rain et al. (2001) using a yeast two-hybrid strategy. Data taken from DIP. GC interations were computed using the *H. pylori* genome (RefSeq:NC_000915)

ND: not determined
